# Supplementary material for: Embryo size regulates the timing and mechanism of pluripotent tissue morphogenesis
Source: Stem Cell Reports. 2020 Oct 8;16(5):1182–96. doi: 10.1016/j.stemcr.2020.09.004 (PMC8185375; doi:10.1016/j.stemcr.2020.09.004)
Supplement: Document S1. Supplemental Experimental Procedures, Figures S1–S4, and Tables S1 and S2 [file mmc1.pdf]

Stem Cell Reports, Volume *16*

Supplemental Information

Embryo size regulates the timing and mechanism of pluripotent tissue morphogenesis

Lorenzo C. Orietti, Viviane Souza Rosa, Francesco Antonica, Christos Kyprianou, William Mansfield, Henrique Marques-Souza, Marta N. Shahbazi, and Magdalena Zernicka-Goetz

SUPPLEMENTARY INFORMATION

Embryo size regulates the timing and mechanism of pluripotent tissue morphogenesis

Lorenzo C. Orietti[1,2#], Viviane Souza Rosa[1,3#], Francesco Antonica[1,4], Christos Kyprianou[1], William Mansfield[5], Henrique Marques-Souza[3], Marta N. Shahbazi[1,6*], Magdalena Zernicka-Goetz[1,2,7*]

SUPPLEMENTARY FIGURE LEGENDS

FIGURE S1: Further characterization of single and double embryos and ESC aggregates. Related to Figure 1 and Figure 3.

Representative images of single and double embryos recovered at the 8-cell stage and cultured *in vitro* to the blastocyst stage (**A**). Cell counts in single and double blastocysts (**B**). Representative image of double embryo at stage **III** showing a multiple lumen phenotype (**C**). Square denote magnified region and arrows indicate multi-lumens in **C.** Distribution and size of ESC aggregates at 24 (**D, E**), 48 (**F, G**), 72 (**H, I**) and 96 (**J, K**) hours after removal of naïve pluripotency factors. Representative image of a multi-layered ESC aggregate at 48 hours stained for F-ACTIN and GM130 (**L**). Calculation of the cell aspect ratio in outer and inner cells in ESC aggregates (**M**). Dotted lines mark cell height and continuous lines mark cell width. Squares denote magnified regions in **L** and arrows indicate GM130. Schematic representation illustrating Golgi localization in outer polarized and inner unpolarized cells (**N**). Number of cells of 2, 4 and 8 cell aggregates 48 (**O**) and 72 (**P**) hours after plating. Number of cells of 8 cells aggregates with single or multiple lumen at 48 hours (**Q**). In panel L Outer cells n=54, inner cells n=57 from 5 aggregates. In panel B single embryos n=16, double embryos n=5. In panels D-K and O-P, number of aggregates is indicated in Figure 3. In panel Q, aggregates with single lumen, n=10 and aggregates with multiple lumen, n=10. Statistical analyses: in panels B, M and Q, Student's *t*-test. ****p<0.0001, **p<0.01. In panels O and P, one-way ANOVA. ****p<0.0001. Dot plots display mean ± S.D. Scale bar 50 μm.

FIGURE S2: The rate of apoptosis increases upon exit from naïve pluripotency. Related to Figure 3.

Representative images of REX1::GFPd2 ESC aggregates cultured in basal medium alone (-2iLIF) (**A**, **B**) or basal medium supplemented with naïve pluripotency factors (+2iLIF) (**C**, **D**) for 72 or 96 hours. Percentage of ESC aggregates with no apoptotic cells (light grey), 1-2 apoptotic cells (green) or more than 3 apoptotic cells (red) in the aggregate at 72 (**E**) and 96 (**F**) hours. Percentage of ESC aggregates of different sizes showing no lumen (grey) or single and multiple lumens (blue) at 72 (**G**) and 96 (**H**) hours. At 72 hours, +2iLIF n=19, -2iLIF n=25. At 96 hours +2iLIF n=16, -2iLIF n=29. 1 independent experiment. Statistical analyses: χ^2 test. ***p<0.001, **p<0.01, *p<0.05, *ns*=not significant. Arrowheads indicate cleaved CASPASE-3 (CASP3) positive cells, dotted lines indicate lumens. Scale bars 50 μm.

FIGURE S3: Lack of basement membrane contact triggers apoptosis of ESCs cultured as aggregates. Related to Figure 3.

The basement membrane provides a survival signal for outside cells in ESC aggregates: Representative images of ESC aggregates cultured in matrigel (control) or in agarose for 24 (**A**), 48 (**B**), or 72 (**C**) hours after removal of naïve pluripotency factors. Arrowheads indicate cleaved CASPASE-3 (CASP3) positive cells, dotted lines indicate lumens. 1 independent experiment. Scale bars 50 μm. Representative images of ESC aggregates cultured in agarose with basal medium supplemented with naïve

pluripotency factors (+2iLIF) or basal medium alone (-2iLIF) (**D**) for 72 hours. Apoptotic index in ESC aggregates cultured in agarose with basal medium +2iLIF or -2iLIF (**E**). +2iLIF n=32, -2iLIF n=31. 3 independent experiments. Scale bars 30 μm. Statistical analyses: Mann Whitney. ****p<0.0001. Arrowheads indicate cleaved CASPASE-3 (CASP3) positive cells. Bar charts display mean ± SEM. Snapshots of representative GFP-PODXL (grey) overexpressing ESC small (**F**), medium (**G**) and large (**H**) aggregates cultured in the presence of SYTOX (green) and subjected to live imaging for 96 hours. Arrowheads indicate increased SYTOX signal, dotted lines indicate lumens. Scale bars 50 μm.

FIGURE S4: Chemical inhibition of apoptosis delays but does not prevent single lumen formation in large ESC aggregates. Related to Figure 4.

Representative images of ESC aggregates cultured in DMSO or with the apoptosis inhibitor Z-VAD-FMK (Z-VAD) for 72 (**A**) or 96 (**B**) hours after removal of naïve pluripotency factors. Percentage of ESC aggregates with no apoptotic cells (light grey), 1-3 apoptotic cells (green) or more than 3 apoptotic cells (red) in the lumen at 72 (**C**) and 96 (**D**) hours. Percentage of ESC aggregates of different sizes with no lumen (grey), single lumens (blue) or multiple lumens (yellow) at 72 (**E**) and 96 (**F**) hours. At 72 hours, DMSO n=21, Z-VAD n=28. At 96 hours DMSO n=36, Z-VAD n=37. 2 independent experiments. Statistical analyses: χ^2 test. ****p<0.0001, ***p<0.001, **p<0.01, *p<0.05, *ns* =not significant. Arrowheads indicate cleaved CASPASE-3 (CASP3) positive cells, dotted lines indicate lumens. Scale bars 50 μm. Classification of BCL-2 overexpressing ESC aggregates into different bins based on size**:** Representative images of control and Bcl-2 overexpressing ESC stained for OCT4 (red) and BCL-2 (grey) (**G**). Distribution and size of apoptosis deficient Bcl-2 overexpressing ESC aggregates at 48 (**H, I**), 72 (**J**, **K**) and 96 (**L**, **M**) hours after removal of naïve pluripotency factors. In panels H-M, number of aggregates is indicated in Figure 4. Dot plots display mean ± S.D. Scale bars 50 μm.

SUPPLEMENTARY TABLES

Table S1: Exclusion criteria for embryos with apoptosis-deficient epiblast at E5.5. Related to Figure 5.

E5.5	Disorganised	Low Chimerism	Single lumen	Multiple lumen	Disorganised EPI*
**Single**	3/12	1/12	5/8	2/8	1/8
**Double**	2/17	3/17	2/12	5/12	5/12

*EPI: epiblast.

Table S2: Exclusion criteria for embryos with apoptosis-deficient epiblast at E6.5. Related to Figure 5.

E6.5	Disorganised	Gastrulating	Low Chimerism	Normal PAC*	Abnormal PAC
**Single**	1/8	0/8	0/8	3/7	4/7
**Double**	1/14	2/14	0/14	0/11	11/11

*PAC: pro-amniotic cavity.

SUPPLEMENTARY MOVIES

MOVIE S1

Representative small ESC aggregate imaged from 24 to 96 hours after removal of naïve pluripotency factors. GFP–PODXL (grey), SYTOX (green). Scale bar 50 μm.

MOVIE S2

Representative medium ESC aggregate imaged from 24 to 96 hours after removal of naïve pluripotency factors. GFP–PODXL (grey), SYTOX (green). Scale bar 50 μm.

MOVIE S3

Representative large ESC aggregate imaged from 24 to 96 hours after removal of naïve pluripotency factors. GFP–PODXL (grey), SYTOX (green). Scale bar 50 μm.

SUPPLEMENTARY EXPERIMENTAL PROCEDURES

Mouse ESC culture

Mouse ESCs were cultured in gelatin coated plates in Fc or N2B27 containing the naïve pluripotency factors 2iLIF (1 µM MEK inhibitor PD0325901 (Stem Cell Institute), 3 µM GSK3 inhibitor CHIR99021 (Stem Cell Institute) and 10 ng ml^{-1} LIF (Stem Cell Institute)) at 37 °C, 5% CO_2, 21% O_2. Fc medium composition was DMEM (41966, Thermo Fisher Scientific), 15% fetal bovine serum (Stem Cell Institute), penicillin–streptomycin (15140122, Thermo Fisher Scientific), GlutaMAX (35050061, Thermo Fisher Scientific), MEM non-essential amino acids (11140035, Thermo Fisher Scientific), sodium pyruvate (11360070, Thermo Fisher Scientific) and 100 µM β-mercaptoethanol (31350-010, Thermo Fisher Scientific). N2B27 was prepared with a 1:1 mix of DMEM F12 (21331-020, Thermo Fisher Scientific) and neurobasal A (10888-022, Thermo Fisher Scientific) plus 1% v/v B27 (10889-038, Thermo Fisher Scientific), 0.5% v/v N2 (homemade), 100 µM β-mercaptoethanol (31350-010, Thermo Fisher Scientific), penicillin–streptomycin (15140122, Thermo Fisher Scientific) and GlutaMAX (35050061, Thermo Fisher Scientific). N2 supplement composition was DMEM F12 medium (21331-020, Thermo Fisher Scientific), 2.5 mg ml^{-1} insulin (I9287, Sigma-Aldrich), 10 mg ml^{-1} Apo-transferrin (T1147, Sigma-Aldrich), 0.75% bovine albumin fraction V (15260037, Thermo Fisher Scientific), 20 µg ml^{-1} progesterone (p8783, Sigma-Aldrich), 1.6 mg ml^{-1} putrescine dihydrochloride (P5780, Sigma-Aldrich) and 6 µg ml^{-1} sodium selenite (S5261, Sigma-Aldrich). Cells were passaged with trypsin-EDTA (25300054, Thermo Fisher Scientific) and routinely tested for contamination by mycoplasma.

E14 wild-type mouse ESCs were derived as previously described (Shahbazi *et al.*, 2017). Briefly, 8-cell stage 129aa mouse embryos were collected from the oviducts of pregnant females and cultured *in vitro* for 24 hours in KSOM supplemented with 2i, followed by 48 hours culture in N2B27 supplemented with 2iLIF. The resulting expanded and hatched blastocysts were plated in individual wells of a 96 well plate with mitomycin-C (M4287, Sigma)-treated mouse embryonic fibroblasts (feeder cells) (ASF-1201, AMS Biotechnology) in Fc medium supplemented with 2iLIF. After 48 hours blastocysts outgrowths were passaged by treatment with trypsin-EDTA for 20 minutes. After two passages in feeder cells, mouse ESCs were routinely cultured in gelatin-coated plates. Rex1::GFPd2 cells were kindly provided by Prof. Austin Smith, Wellcome - MRC Cambridge Stem Cell Institute.

Cloning

- PB TetO GFP-Podocalyxin Hygro: a GFP-Podocalyxin pDONR221construct (Shahbazi et al., 2017) was used to subclone GFP-Podocalyxin into a PB TetO hygro destination vector (kind gift of Dr. Jose Silva, MRC Cambridge Stem Cell Institute) using an LR clonase II (11791-100, Life technologies).
- PB rtTA3 Zeo: a pEnt L1L3-EF1a-rtTA3 (addgene plasmid #27106) was used as a template to amplify rtTA3 by PCR using the following primers:

attB1-rtTA3 FW:

5'-
GGGGACAAGTTTGTACAAAAAAGCAGGCTTCACCATGTCTAGACTGGACAAGA
GCAAAGTC-3'

attB2-rtTA3 RV:

5'-GGGGACCACTTTGTACAAGAAAGCTGGGTCTTACCCGGGGAGCATGTCAA-3'

The rtTA3 fragment containing attB sites was cloned into a pDONR221 vector using a BP clonase II enzyme (11789-100, Life Technologies), and further subcloned into a PB Zeo plasmid (kind gift of Jose Silva, MRC Cambridge Stem Cell Institute) using the LR clonase II.

- PB Bcl-2 Hygro: Bcl-2 was amplified by PCR from mouse cDNA using the following primers:

Bcl2 FW: 5'-ATGGCGCAAGCCGGGAG-3'

Bcl2 RV: 5'-TCACTTGTGGCCCAGGTATGCAC-3'

In a subsequent PCR amplification attB sites were added using the following primers:

attB1-Bcl-2 FW:

5'-GGGGACAAGTTTGTACAAAAAAGCAGGCTTCACCATGGCGCAAGCCGGGAG-3'

attB2-Bcl2 RV:

5'-GGGGACCACTTTGTACAAGAAAGCTGGGTCTCACTTGTGGCCCAGGTATGC-3'

The Bcl-2 fragment containing attB sites was cloned into a pDONR221 vector using a BP clonase II enzyme, and further subcloned into a PB Hygro plasmid (kind gift of Jose Silva, MRC Cambridge Stem Cell Institute) using the LR clonase II.

Cell transfection

E14 mouse ESCs were transfected using Lipofectamine 3000 (L3000001, Thermo Fisher Scientific) in agreement with the manufacturer's recommendations. Briefly, 50.000 were cells seeded in a 24 well plate dish in Fc medium supplemented with 2iLIF the day prior to the transfection. Transfection was done with 0.3 µg of the plasmid(s) containing the gene(s) of interest and 0.2 µg of a plasmid expressing a PiggyBac transposase (kind gift of Prof. Jose Silva, MRC Cambridge Stem Cell Institute). Transfected cells were selected using the relevant antibiotics as outlined below:

- Doxycycline inducible GFP-PODOCALYXIN expressing mouse ESCs: cells were selected with 200 µg mL-1 of hygromycin B (10687010, Thermo Fisher Scientific) and 100 µg mL-1 of zeocin (ant-zn-1, Invivogen).

- BCL-2 overexpressing mouse ESCs: cells were selected with 200 µg mL-1 of hygromycin B. Experiments were performed with a mixed population of cells as individual clones were not picked.

H2B-GFP expressing mouse ESCs have been described elsewhere (Shahbazi *et al.*, 2017)

Live imaging

GFP-Podocalyxin expression was triggered by adding 1 µg mL^{-1} doxycycline hyclate (D9891, Sigma) to the medium. SYTOX Orange (S11368, Thermo Fisher Scientific) was used according to the manufacturer's instructions. Imaging was carried out on a Leica SP8 inverted confocal microscope (Leica Microsystem) using a Leica Fluotar VISIR 0.95 NA x25 objective. Time points were taken every 60 minutes between 24 and 96 hours after 2iLIF withdrawal with a z-step of 2.5 µm. Cells were imaged in a humidified chamber at 37 °C and 5% CO_2.

Immunostaining

Embryos and mouse ESCs were fixed in 4% Paraformaldehyde (11586711, Electron Microscopy Sciences) for 20 minutes at room temperature and permeabilized for 20 minutes in PBS 0.5% Triton X-100. Samples were incubated overnight at 4°C in blocking buffer (10% filtered fetal bovine serum, 0.1% Triton X-100 in PBS) with primary antibodies. Incubation with secondary antibodies was carried out for 2 hours at room temperature in blocking buffer. The following primary antibodies were used: Cleaved CASPASE-3 (9664S, Cell Signaling Technology) at 1:300, GM130 (610822, BD Biosciences) at 1:300, OCT4 (sc-5279, Santa Cruz Biotechnology) at 1:300, NANOG (ab80892, Abcam) at 1:300, CDX2 (MU392-UC, Launch Diagnostics) at 1:300, SOX17 (AF1924, R&D Systems) at 1:300, PODOCALYXIN (MAB1556, R&D Systems) at 1:500, BCL-2 (ab182858, Abcam) at 1:300, GFP (GF090R, Fine Chemical Products Ltd) at 1:500, COLLAGEN IV (AB769, Millipore) at 1:300. Conjugated secondary antibodies from Thermo Fisher Scientific were used at 1:500. F-ACTIN was visualized using Alexa Fluor488-phalloidin (A12379, ThermoFisher Scientific) or Alexa Fluor647-phalloidin (A22287, ThermoFisher Scientific) at 1:500. Nuclei were stained with DAPI (D3571, Life Technologies) at 1:500.

Imaging and Image analyses

Scanning was carried out on a Leica SP5 or SP8 inverted confocal microscope (Leica Microsystem) using a Leica HC PL APO CS2 40x or a Leica Fluotar VISIR 0.95 NA 25x objective. The software Fiji (Schneider et al., 2012) was used for image processing and analysis. Cell numbers in pre- and post-implantation embryos and in mouse ESC aggregates were manually counted in Fiji using the "Multi-point" tool. In E4.5 and E5.5 embryos all epiblast cells were counted by selecting individual nuclei across the different Z-planes. Quantifications shown in panels 1J, S1O-Q and 5K were

done on a single representative Z-plane. The apoptotic index was calculated by dividing the number of cleaved CASPASE-3 positive cells by the total number of epiblast cells in embryo analyses and by the total number of cells in ESC aggregates cultured in agarose. For the analysis of apoptosis in mouse ESC aggregates, the number of cleaved CASPASE-3 positive cells within the lumen was manually counted in Fiji. For the analyses shown in Figure S2E-F the total number of apoptotic cells in a representative single plane was counted, since aggregates cultured in naïve pluripotent conditions do not have lumens. For the classification of mouse ESC aggregates into different bins, for each experiment the area of individual aggregates in the maximum projection was measured in Fiji by manual drawing using the "Measure, Area" tool. Mouse ESC aggregates within the percentiles 1-33, 33-66 and 66-100 were considered small, medium and large respectively.

Statistical analyses

Statistical analyses were performed using GraphPad. Sample size for embryo-ESC chimera experiments was determined using power calculations for a χ^2 test (α=0.05, 1-β=0.95) using G*Power. In the rest of experiments sample size was determined based on previous experimental experience. Investigators were not blinded to group allocation and embryos were randomly allocated to control and experimental groups. Exclusion criteria for post-implantation embryos are stated in the text and summarised in Table S1 and S2. Qualitative data are presented as percentages of total events, contingency tables with raw event counts were analysed with a χ^2 test. Quantitative data are presented as mean ± S.D. and were analysed for normality with a D'Agostino-Pearson normality test. If passed, a two-tailed unpaired Student's *t*-test was used while for non-Gaussian distributions a Mann-Whitney-*U*-test was performed. To compare multiple groups an ANOVA test with a Tukey's multiple comparison test was performed. For linear correlation analyses, the Pearson correlation coefficient was computed.

FIGURE S1

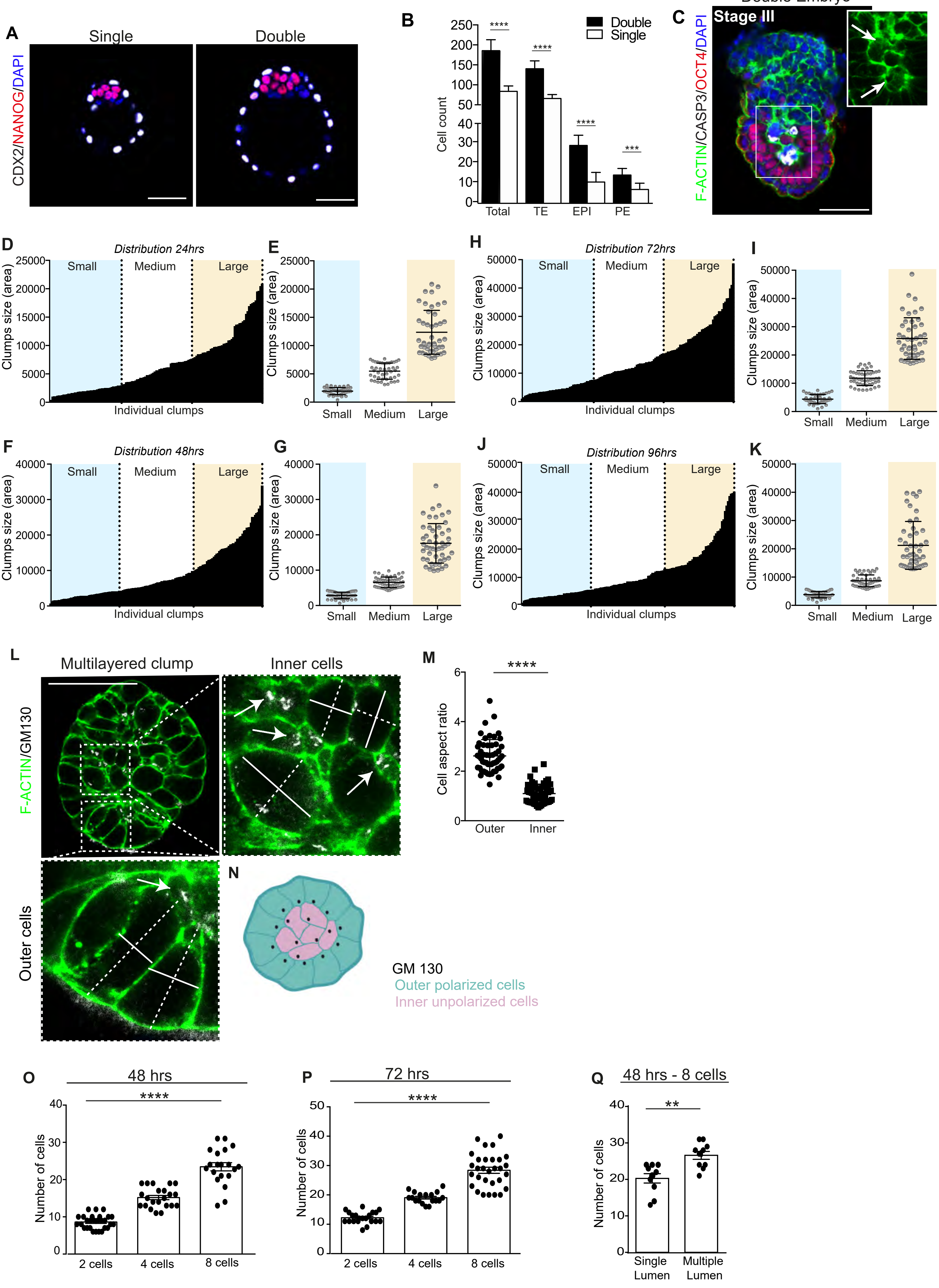

A
Single
Double
CDX2/NANOG/DAPI
B
Double
Single
Cell count
Total
TE
EPI
PE
C
Double Embryo
Stage III
F-ACTIN/CASP3/OCT4/DAPI
D
Distribution 24hrs
Small
Medium
Large
Clumps size (area)
Individual clumps
E
H
Distribution 72hrs
I
F
Distribution 48hrs
G
J
Distribution 96hrs
K
L
Multilayered clump
Inner cells
F-ACTIN/GM130
Outer cells
M
Cell aspect ratio
Outer
Inner
N
GM 130
Outer polarized cells
Inner unpolarized cells
O
48 hrs
Number of cells
2 cells
4 cells
8 cells
P
72 hrs
Q
48 hrs - 8 cells
Single Lumen
Multiple Lumen

FIGURE S2

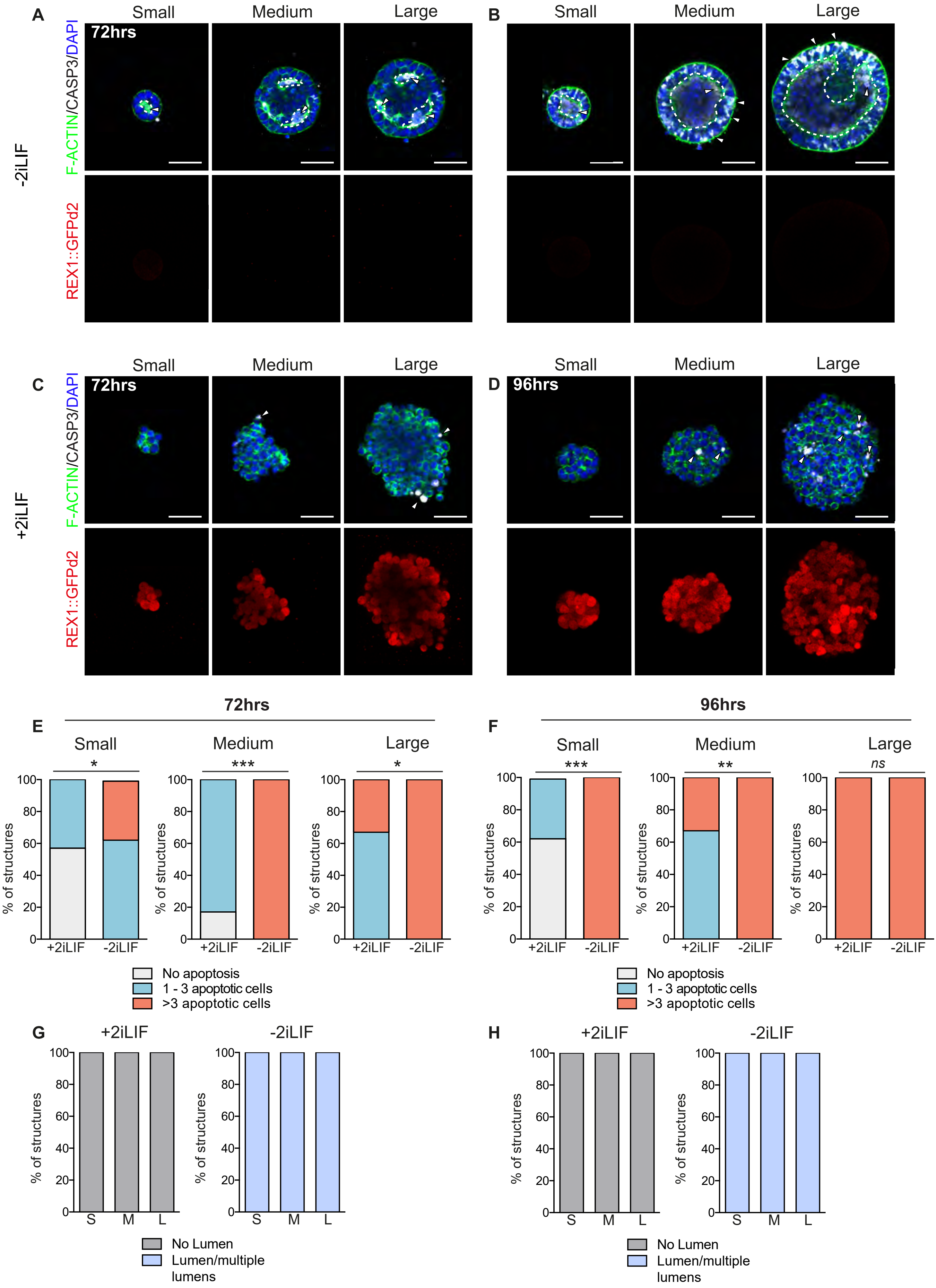
A
Small
Medium
Large
72hrs
-2iLIF
F-ACTIN/CASP3/DAPI
REX1::GFPd2
B
C
+2iLIF
D
96hrs
72hrs
96hrs
E
F
*
***
**
ns
% of structures
+2iLIF
-2iLIF
No apoptosis
1 - 3 apoptotic cells
>3 apoptotic cells
G
H
S
M
L
No Lumen
Lumen/multiple lumens

FIGURE S3

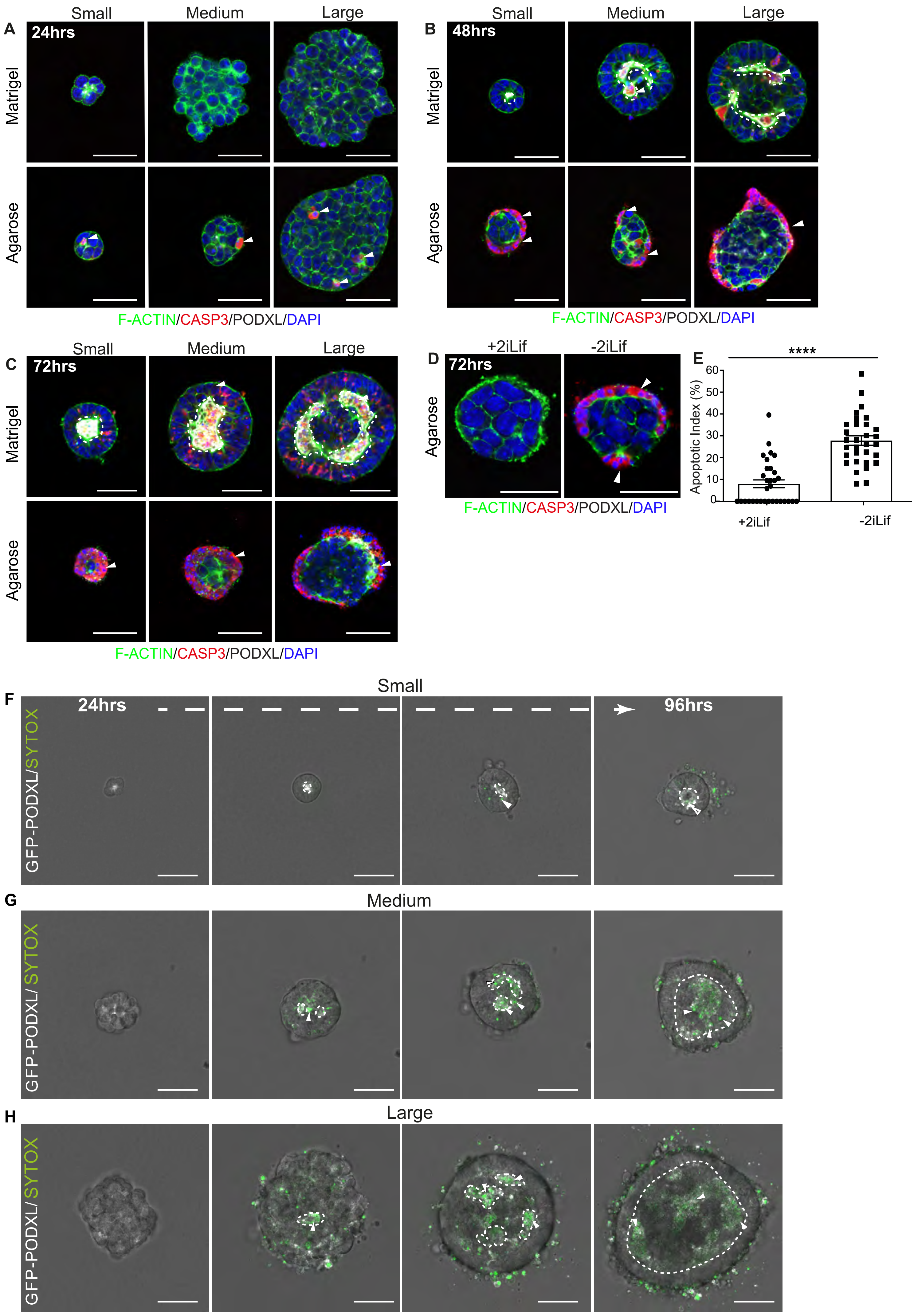
A
24hrs
Small
Medium
Large
Matrigel
Agarose
F-ACTIN/CASP3/PODXL/DAPI
B
48hrs
Small
Medium
Large
Matrigel
Agarose
F-ACTIN/CASP3/PODXL/DAPI
C
72hrs
Small
Medium
Large
Matrigel
Agarose
F-ACTIN/CASP3/PODXL/DAPI
D
72hrs
+2iLif
-2iLif
Agarose
F-ACTIN/CASP3/PODXL/DAPI
E
****
Apoptotic Index (%)
0
10
20
30
40
50
60
+2iLif
-2iLif
F
Small
24hrs
96hrs
GFP-PODXL/SYTOX
G
Medium
GFP-PODXL/ SYTOX
H
Large
GFP-PODXL/ SYTOX

FIGURE S4

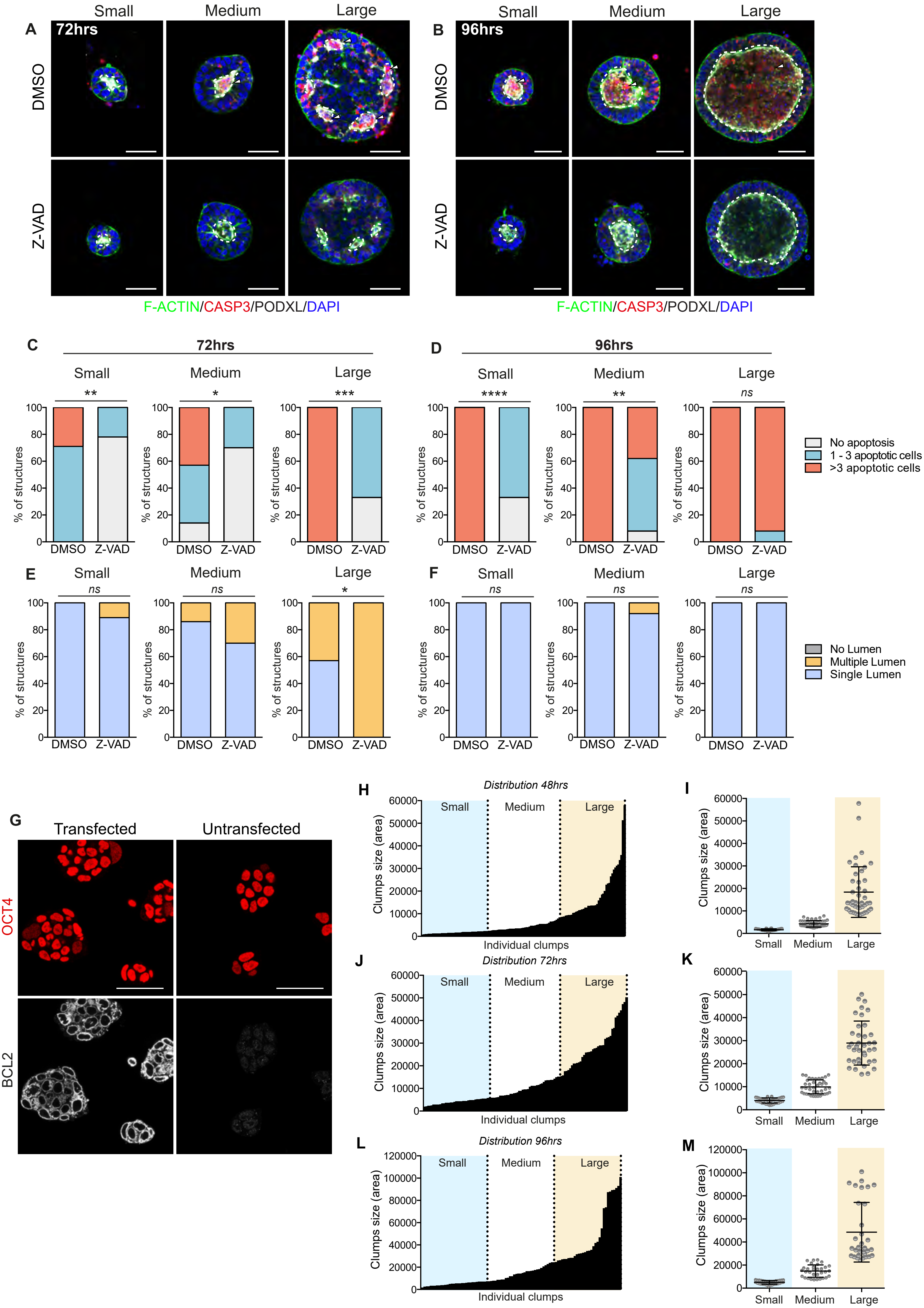

A
B
Small
Medium
Large
72hrs
96hrs
DMSO
Z-VAD
F-ACTIN/CASP3/PODXL/DAPI
C
72hrs
D
96hrs
**
*
***
****
**
ns
% of structures
DMSO
Z-VAD
No apoptosis
1 - 3 apoptotic cells
>3 apoptotic cells
E
F
No Lumen
Multiple Lumen
Single Lumen
G
Transfected
Untransfected
OCT4
BCL2
H
Distribution 48hrs
I
J
Distribution 72hrs
K
L
Distribution 96hrs
M
Clumps size (area)
Individual clumps
