# Supplementary material for: Embryo size regulates the timing and mechanism of pluripotent tissue morphogenesis
Source: Stem Cell Reports. 2020 Oct 8;16(5):1182–96. doi: 10.1016/j.stemcr.2020.09.004 (PMC8185375; doi:10.1016/j.stemcr.2020.09.004)
Supplement: Document S2. Article plus Supplemental information [file mmc5.pdf]

Stem Cell Reports
Article
OPEN ACCESS

Embryo size regulates the timing and mechanism of pluripotent tissue morphogenesis

Lorenzo C. Orietti,[1,2,8] Viviane Souza Rosa,[1,3,8] Francesco Antonica,[1,6] Christos Kyprianou,[1] William Mansfield,[4] Henrique Marques-Souza,[3] Marta N. Shahbazi,[1,7,*] and Magdalena Zernicka-Goetz[1,2,5,*]

[1]Mammalian Embryo and Stem Cell Group, University of Cambridge, Department of Physiology, Development and Neuroscience, Downing Street, Cambridge CB2 3DY, UK
[2]Centre for Trophoblast Research, University of Cambridge, Cambridge, UK
[3]Department of Biochemistry and Tissue Biology, State University of Campinas, CP 6109, 13083-970 Campinas, SP, Brazil
[4]Wellcome – MRC Cambridge Stem Cell Institute, Jeffrey Cheah Biomedical Centre Cambridge Biomedical Campus, Puddicombe Way, Cambridge CB2 0AW, UK
[5]California Institute of Technology (Caltech), Division of Biology and Biological Engineering, Pasadena, CA 91125, USA
[6]Present address: Armenise-Harvard Laboratory of Brain Disorders and Cancer, Department of Cellular, Computational and Integrative Biology CIBIO, University of Trento, Via Sommarive 9, 38123 Trento, Italy
[7]Present address: MRC Laboratory of Molecular Biology, Francis Crick Avenue, Cambridge Biomedical Campus, Cambridge CB2 0QH, UK
[8]These authors contributed equally
*Correspondence: mshahbazi@mrc-lmb.cam.ac.uk (M.N.S.), mz205@cam.ac.uk (M.Z.-G.)
https://doi.org/10.1016/j.stemcr.2020.09.004

SUMMARY

Mammalian embryogenesis is a paradigm of regulative development as mouse embryos show plasticity in the regulation of cell fate, cell number, and tissue morphogenesis. However, the mechanisms behind embryo plasticity remain largely unknown. Here, we determine how mouse embryos respond to an increase in cell numbers to regulate the timing and mechanism of embryonic morphogenesis, leading to the formation of the pro-amniotic cavity. Using embryos and embryonic stem cell aggregates of different size, we show that while pro-amniotic cavity formation in normal-sized embryos is achieved through basement membrane-induced polarization and exocytosis, cavity formation of increased-size embryos is delayed and achieved through apoptosis of cells that lack contact with the basement membrane. Importantly, blocking apoptosis, both genetically and pharmacologically, alters pro-amniotic cavity formation but does not affect size regulation in enlarged embryos. We conclude that the regulation of embryonic size and morphogenesis, albeit concomitant, have distinct molecular underpinnings.

INTRODUCTION

Early mammalian development is regulative, as embryos are able to compensate for perturbations to the normal developmental program to ensure the formation of a viable organism (Lawrence and Levine, 2006; Tarkowski and Wroblewska, 1967). When, for example, two embryos are aggregated together to generate double-sized embryos, they produce pups of normal size (Mintz, 1965; Tarkowski, 1961), albeit at lower efficiency. This result means that embryos can sense deviations from the normal number of cells and regulate their size accordingly. It has been shown that such regulation of embryo size occurs after implantation and before gastrulation through the lengthening of the cell cycle (Buehr and Mclaren, 1974; Lewis and Rossant, 1982). However, how an increased number of cells affects the mechanism of embryonic morphogenesis and its timing remains unknown.

At implantation, mouse and human embryos comprise three cell lineages: embryonic epiblast that will give rise to the future organism; extra-embryonic primitive endoderm that will give rise to the yolk sac; and trophectoderm that will build the placenta. After embryo implantation, these three lineages interact to undertake the first morphogenetic step, remodeling of the epiblast from a group of apolar cells to a polarized epithelium that lines the incipient pro-amniotic cavity (Bedzhov and Zernicka-Goetz, 2014). This is an essential event for the establishment of the body plan and it becomes accomplished by embryonic day 5.0 (E5.0) (Shahbazi and Zernicka-Goetz, 2018; Sheng, 2015).

Studies using embryoid bodies derived from embryonic stem cells (ESCs) as a model for pro-amniotic cavity formation suggested that formation of the lumen entails death of the cells located in the center of the tissue by apoptosis (Coucouvanis and Martin, 1995). However, direct observations of mouse and human embryos developing both *in vivo* and *in vitro* revealed that pro-amniotic cavity formation does not require cell death but takes place through cell polarization, apical membrane repulsion, and exocytosis (Bedzhov and Zernicka-Goetz, 2014; Shahbazi et al., 2016), a process known as hollowing (Datta et al., 2011). During hollowing, embryonic cells establish apicobasal polarity in response to the surrounding basement membrane produced by the primitive endoderm. The resulting rosette of polarized cells undergoes lumenogenesis, which is regulated by the pluripotency network (Bedzhov and Zernicka-Goetz, 2014; Neagu et al., 2020; Shahbazi et al., 2017).

 © 2020 The Authors.
This is an open access article under the CC BY license (http://creativecommons.org/licenses/by/4.0/).

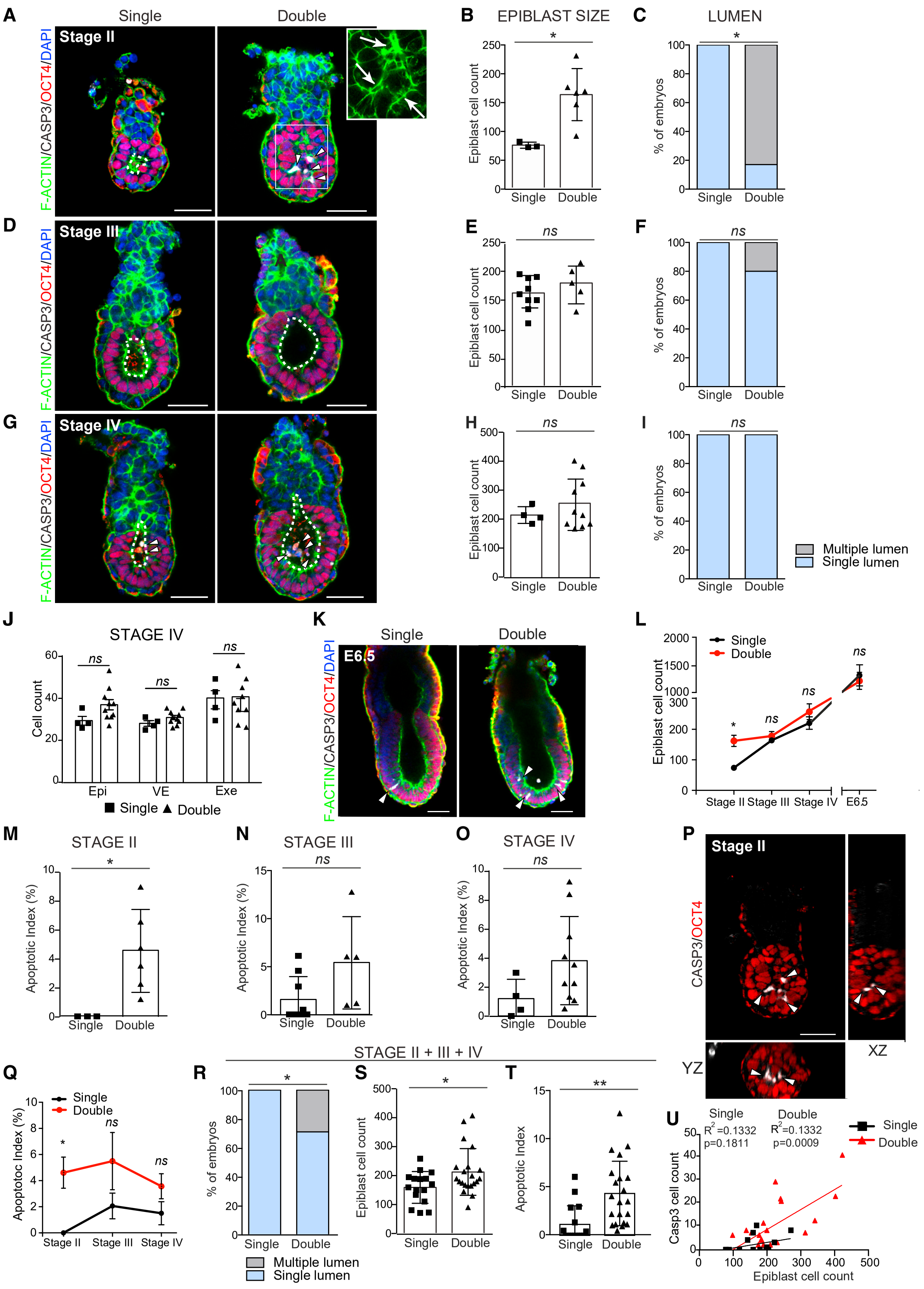

(legend on next page)

The mechanism underpinning lumen formation was confirmed *in vitro* by embedding a small number of ESCs into Matrigel as an exogenous source of basement membrane to induce polarization and lumenogenesis (Bedzhov and Zernicka-Goetz, 2014). This experiment raises the follow-up question of whether cell numbers regulate the timing and mechanism of embryo morphogenesis.

Here, we address this question by generating double-sized embryos and ESC aggregates of different sizes as two complementary model systems. To our knowledge, our results provide the first evidence for a size-dependent mechanism of pro-amniotic cavity formation in the mouse embryo, in agreement with the regulative nature of mammalian development.

RESULTS

Double Embryos Display Greater Apoptosis and Delayed Pro-amniotic Cavity Formation

It is well known that when two pre-implantation embryos are aggregated, they develop into double-sized blastocysts. Subsequent cell-cycle lengthening during early post-implantation development leads to normal-sized gastrulae (Buehr and Mclaren, 1974; Lewis and Rossant, 1982). However, whether embryo size regulates the timing and mechanism of embryo morphogenesis remains unknown. To address this question, we aggregated two pre-compacted 8-cell stage embryos together to form so-called double embryos and followed their subsequent development *in vitro* and *in vivo*. We first confirmed that at the blastocyst stage, double embryos displayed twice the normal number of cells in all three lineages: the epiblast, the primitive endoderm, and trophectoderm (Figures S1A and S1B). To follow embryonic development *in vivo*, we transferred double and control (single) blastocysts into pseudo-pregnant recipients and recovered them at early post-implantation stages. To account for the asynchrony within each litter and across different experiments, we scored embryos according to their morphological features (Christodoulou et al., 2018).

At approximately E5.25 (stage II) (Christodoulou et al., 2018), the epiblast of single embryos comprised polarized cells surrounding an incipient luminal cavity (Figures 1A and 1C). In contrast, the epiblast of double embryos had twice the normal number of cells, which were organized in multiple layers and displayed multiple incipient lumens (Figures 1A–1C), indicating that embryonic size was not yet regulated and the pro-amniotic cavity was not yet formed. By E5.5 (stage III) all single embryos had opened a central lumen (Figure 1D, dotted line), while 20% of double embryos still displayed a multi-lumen phenotype (Figures 1F and S1C). At this stage, single and double embryos had similar cell numbers in the epiblast (Figure 1E), demonstrating that size regulation was completed by E5.5, in agreement with previous results showing that size reduction is achieved before gastrulation (Buehr and Mclaren, 1974; Lewis and Rossant, 1982). By E5.75 (stage IV), single and double embryos displayed a cup-shaped epiblast with a single expanded cavity extending toward the extra-embryonic ectoderm compartment (Figures 1G–1I).

Figure 1. Double Embryos Display a Higher Apoptotic Rate and Delayed Lumenogenesis Compared with Single Embryos

(A–Q) Representative images of single and double embryos at stages II (A), III (D), and IV (G). Epiblast cell counts of single and double embryos at stages II (B), III (E), and IV (H). Percentages of single and double embryos showing a single lumen (blue) or multiple lumen (gray) at stages II (C), III (F), and IV (I). Epiblast (Epi), visceral endoderm (VE), and extra-embryonic ectoderm (Exe) cell counts in single and double embryos at stage IV (J). Representative images of single and double embryos recovered at E6.5 (K). Epiblast cell counts in single and double embryos between stage II and E6.5 (L). Apoptotic indexes in the epiblast of single and double embryos at stages II (M), III (N), and IV (O). Orthogonal views of the double embryo shown in (A) (P). Summary of apoptotic indexes in single and double embryos during stages II–IV (Q).

(R) Analysis of pro-amniotic cavity formation using time of recovery as staging criteria. Embryos were recovered at 10 a.m. 5.5 days after the positive plug was found. Percentages of single and double embryos with a single lumen (blue) or multiple lumen (gray) are shown.

(S) Epiblast cell counts of single and double embryos using time of recovery as staging criteria. Embryos were recovered at 10 a.m. 5.5 days after the positive plug was found.

(T) Apoptotic index in the epiblast of single and double embryos using time of recovery as staging criteria. Embryos were recovered at 10 a.m. 5.5 days after the positive plug was found.

(U) Correlation between total number of OCT4-positive cells (Epiblast cell count) and number of epiblast cells positive for cleaved CASPASE-3 (Casp3) in single and double embryos between stages II and IV.

In (B) to (I) and (M) to (O), total single embryos n = 16, double embryos n = 21; stage II, single embryos n = 3, double embryos n = 6; stage III, single embryos n = 9, double embryos n = 5; stage IV, single embryos n = 4, double embryos n = 10; seven independent experiments. At E6.5 single embryos n = 4, double embryos n = 3; one independent experiment. In (B), (E), (H), (J), (L), (M), (N), (O), (Q), (S), and (T), statistical analyses: Student's t test. $^{**}p < 0.01$, $^{*}p < 0.05$; *ns*, not significant. In (C), (F), (I), and (R), statistical analyses: χ^2 test. $^{*}p < 0.05$; *ns*, not significant. In (U), Pearson linear correlation. Bar charts display mean ± SD. Squares denote magnified regions and arrows indicate multi-lumens in (A). Arrowheads indicate cleaved CASPASE-3 (CASP3)-positive cells in the epiblast, dotted lines indicate pro-amniotic cavity. Scale bars, 50 μm.

We found no morphological differences between single and double embryos and a similar number of cells in both embryonic and extra-embryonic tissues (Figures 1H and 1J), indicating that by this stage size regulation and pro-amniotic cavity formation had both been attained. We obtained similar findings upon embryo recovery at E6.5 (Figures 1K–1L). These results indicate that pro-amniotic cavity formation is delayed in double embryos and happens concomitantly with size regulation (between E5.25 and E5.5; stages II and III).

Since it has been shown that increasing the density of Madin-Darby canine kidney (MDCK) cells results in cysts that open a lumen through apoptosis rather than cell polarization (Martin-Belmonte et al., 2008), we hypothesized that increasing the number of epiblast cells might also lead to an increase in the rate of apoptosis. To address this possibility, we stained double and single embryos for the apoptotic marker cleaved CASPASE-3 (Banfalvi, 2017; Slee et al., 1999). We found that whereas single embryos lacked apoptotic cells at E5.25, in agreement with previous reports (Bedzhov and Zernicka-Goetz, 2014), approximately 5% of cells were apoptotic at that time in double embryos (Figure 1M). Interestingly, activation of the apoptotic pathway was restricted to those inner cells, which were not in contact with the underlying basement membrane (Figures 1A and 1P [arrowheads]). At subsequent stages, we did not observe significant differences in the apoptotic index between single and double embryos (Figures 1N, 1O, and 1Q). These results indicate that there is a specific increase in apoptosis in double embryos prior to pro-amniotic cavity formation.

As an alternative to using morphological stages, we also analyzed our data with respect to time of embryo recovery, as previously reported (Lewis and Rossant, 1982, Buehr and Mclaren, 1974). These analyses confirmed the multi-lumen phenotype, the delay in pro-amniotic cavity formation, and the increase in the number of apoptotic cells in double embryos in comparison with single embryos (Figures 1R–1T). Moreover, we found a strong positive correlation between the total number of epiblast cells (expressing pluripotency marker $OCT4^{+}$) and the number of epiblast cells positive for cleaved CASPASE-3 (Figure 1U). These results suggest that the extent of apoptosis in the epiblast of early post-implantation embryos depends on the epiblast size.

To better understand the relation between polarization, lumenogenesis, and apoptosis in double embryos, we next used isolated inner cell masses (ICMs). These comprise both epiblast and primitive endoderm, and recapitulate the mechanism of amniotic cavity formation in the absence of trophectoderm derivatives: primitive endoderm cells secrete laminin, which triggers epiblast polarization and lumenogenesis (Bedzhov and Zernicka-Goetz, 2014; Li et al., 2003). To isolate ICMs, we subjected embryos at the blastocyst stage to immunosurgery, which eliminated the outer trophectoderm layer (Solter and Knowles, 1975), and cultured the isolated ICMs through the pre-to post-implantation transition in hanging drops (Figure 2A). After 24 h we observed that epiblast cells in single ICMs established apicobasal polarity and were organized into rosette-like structures, although the majority had not initiated lumenogenesis (Figures 2B and 2D). In contrast, cultured double ICMs showed twice as many cells in the epiblast, which was multi-layered and disorganized (Figures 2B and 2C). Moreover, while single ICMs showed no apoptosis, the apoptotic index of double ICMs was significantly higher (Figures 2B and 2E [arrowheads]). We also found that outer epiblast cells in contact with the basement membrane showed polarization, as assessed by the positioning of the Golgi identified through subunit GM130 staining, and displayed a columnar morphology, in contrast to inner cells (Figures 2F–2H). The numbers of inner cells positively correlated with the total number of epiblast cells (Figures 2I–2K), indicating that the presence of inner cells is likely a consequence of the increased cell numbers. By 48 h, both control and double ICMs had initiated lumenogenesis (Figures 2L and 2N). At this stage, the epiblast size and the incidence of apoptosis were still significantly higher in double ICMs (Figures 2M and 2O). By 72 h, the number of epiblast cells in single and double ICMs was not significantly different (Figures 2P and 2Q). These results indicate that size regulation of the epiblast can take place *in vitro* in the absence of trophectoderm cells. At this stage, both single and double ICMs presented a single lumen containing apoptotic cells (Figures 2R and 2S), in agreement with our results in intact embryos. In conclusion, using a combination of *in vivo* and *in vitro* approaches, our results indicate that increasing embryo size leads to the formation of a multi-layered and multi-lumen epiblast and a delay in pro-amniotic cavity formation, which is associated with apoptosis of inner epiblast cells that lack contact with the basement membrane.

Lumen Formation in Double Embryos Can Be Modeled with ESCs

To gain further insight into the relationship between cell numbers and the mechanism of lumenogenesis, we sought to use three-dimensional (3D) aggregates of mouse ESCs as a model of epiblast of varying size. We previously showed that upon exit from naive pluripotency, single ESCs embedded in Matrigel are able to polarize and open a lumen through membrane repulsion, a process that recapitulates lumenogenesis *in vivo* (Bedzhov and Zernicka-Goetz, 2014; Shahbazi et al., 2017). To mimic the double-sized epiblast, we increased the number of cells and seeded different amounts of ESCs onto non-adherent dishes in naive pluripotent conditions, which allowed cells

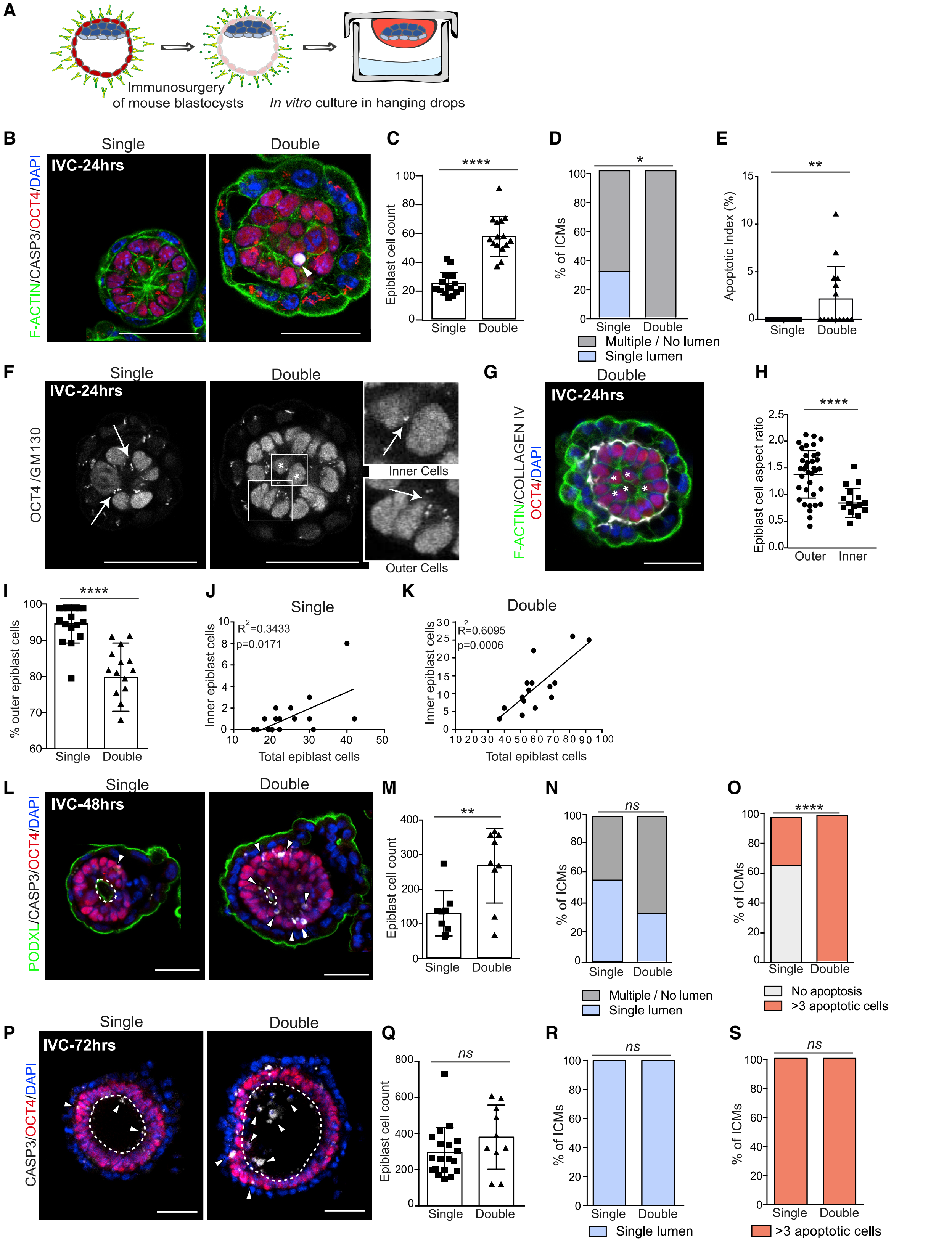

(legend on next page)

to form aggregates of different sizes while maintaining their naive pluripotent state.

After 24 h we collected the aggregates of different sizes, classified them into small, medium, and large based on their size (Figures S1D–S1K), and plated them into a 3D Matrigel culture in the absence of naive pluripotency factors (Figure 3A). Throughout the following 96 h, we scored the presence or absence of lumens (based on the staining of the luminal protein PODOCALYXIN and F-ACTIN) and the incidence of apoptosis. We found that after 24 h, small aggregates displayed one layer of cells in direct contact with the basement membrane, apical constriction, and accumulation of F-ACTIN and PODOCALYXIN (Figure 3B, left panel). In contrast, medium and large aggregates exhibited multiple cell layers showing scattered foci enriched in F-ACTIN and PODOCALYXIN (Figure 3B, middle and right panels). At this stage, only a few small- and medium-sized aggregates had already formed a cavity, while we did not observe a clear lumen in the majority of structures (Figure 3D). We did not detect apoptosis irrespective of aggregate size (Figure 3C), suggesting that activation of the apoptotic pathway does not take place immediately after naive pluripotency exit. In support of this result, we observed that the incidence of apoptosis was very low in mouse ESC aggregates cultured in 3D under naive pluripotent conditions (Figures S2A–S2F). Preserving the naive state also abolished lumen formation (Figures S2G and S2H), as previously described (Shahbazi et al., 2017).

After 48 h in culture, nearly 90% of structures formed from small ESC aggregates showed a single cavity (Figures 3E and 3G) and were devoid of dead cells (Figure 3F), in agreement with our previous work showing that single ESCs cultured in Matrigel undergo lumenogenesis via hollowing (Bedzhov and Zernicka-Goetz, 2014). By contrast, the vast majority of large structures contained at least one apoptotic cell (Figures 3E [arrowheads] and 3F) and displayed mostly multiple lumens (Figure 3G). Staining of the Golgi subunit GM130 and assessment of the cell aspect ratio in multi-layered aggregates confirmed that the outside cells in contact with the basement membrane established apicobasal polarity, indicating that multiple lumen formation relies on polarization and hollowing (Figures S1L–S1N).

By 72 h, we detected a further increase in apoptosis in medium and large structures, which was accompanied by the emergence of single lumens in these groups (Figures 3H–3J). We also observed a modest increase in apoptosis in the small aggregate group at this time, which may be attributable to cells becoming confluent during prolonged *in vitro* culture, as their size plateaued after 3 days (data not shown). By 96 h, all aggregates displayed single lumens irrespective of their size (Figures 3K and 3M), and higher levels of apoptosis were detected in the structures derived from medium and large aggregates (Figure 3L). To test whether the activation of apoptosis was due to lack of interactions with the basement membrane, we cultured ESC aggregates in agarose, in either the presence or absence of naive pluripotency factors. Under these conditions lumens did not form, and we observed widespread activation of CASPASE-3 only in the absence of naive pluripotency factors (Figure S3A–S3E). These results indicate that lack of basement membrane contact triggers cell death in ESC aggregates upon naive pluripotency exit.

To further validate these results, we manually picked ESC aggregates of a defined size (two, four, or eight cells) and allowed them to develop in 3D Matrigel. We first confirmed that the difference in size was still maintained after 48 and 72 h of culture (Figures S1O and S1P), then assessed the levels of apoptosis and the formation of the lumen. We found that 8-cell aggregates had a higher incidence of

Figure 2. Absence of Trophectoderm Cells Does Not Prevent Size Regulation and Lumenogenesis of the Epiblast *In Vitro*
(A) Schematic representation of experimental setup for *in vitro* culture of ICMs.
(B–S) Representative images of single and double ICMs cultured *in vitro* (IVC) for 24 (B), 48 (L), and 72 (P) h. Epiblast cell counts of single and double ICMs cultured *in vitro* for 24 (C), 48 (M), and 72 (Q) h. Percentages of *in vitro* cultured single and double ICMs showing a single lumen (blue) or multiple/no single lumen (gray) at 24 (D), 48 (N), and 72 (R) h. Apoptotic indexes in the epiblast of single and double ICMs cultured *in vitro* for 24 h (E) and percentages of *in vitro* cultured single and double ICMs showing no apoptosis or more than three apoptotic cells at 48 h (O) and 72 h (S). Single and double ICMs cultured *in vitro* stained for OCT4 and for the apical Golgi marker GM130 (F). Representative image of an *in vitro* cultured double ICM for 24 h stained for the basement membrane component COLLAGEN IV (G). Cell aspect ratio in outer and inner epiblast cells of *in vitro* cultured double ICMs (H). Proportion of outer epiblast cells in single and double ICMs cultured *in vitro* (I). Correlation between inner and total number of epiblast cells in single (J) and double (K) ICMs cultured *in vitro* for 24 h.
In (C) to (E) and (I) to (K), single ICMs n = 16, double ICMs n = 15. In (M) to (O), single ICMs n = 9, double ICMs n = 9. In (Q) to (S), single ICMs n = 19, double ICMs n = 10. Two independent experiments. In (H), outer cells n = 38, inner cells n = 17. In (C), (H), (I), (M), and (Q), statistical analyses: Student's t test. ****$p < 0.0001$, **$p < 0.01$; *ns*, not significant. In (E), statistical analysis: Mann-Whitney U test. **$p < 0.01$. In (D), (N), (O), (R), and (S), statistical analysis: χ^2 test. ****$p < 0.0001$, *$p < 0.05$; *ns*, not significant. In (J) and (K), Pearson linear correlation. Bar charts display mean ± SD. Squares denote magnified regions and arrows indicate GM130 localization. Arrowheads indicate cleaved CASPASE-3 (CASP3)-positive cells in the epiblast, dotted lines indicate pro-amniotic cavity, asterisks indicate inner cells in double ICMs. Scale bars, 50 μm.

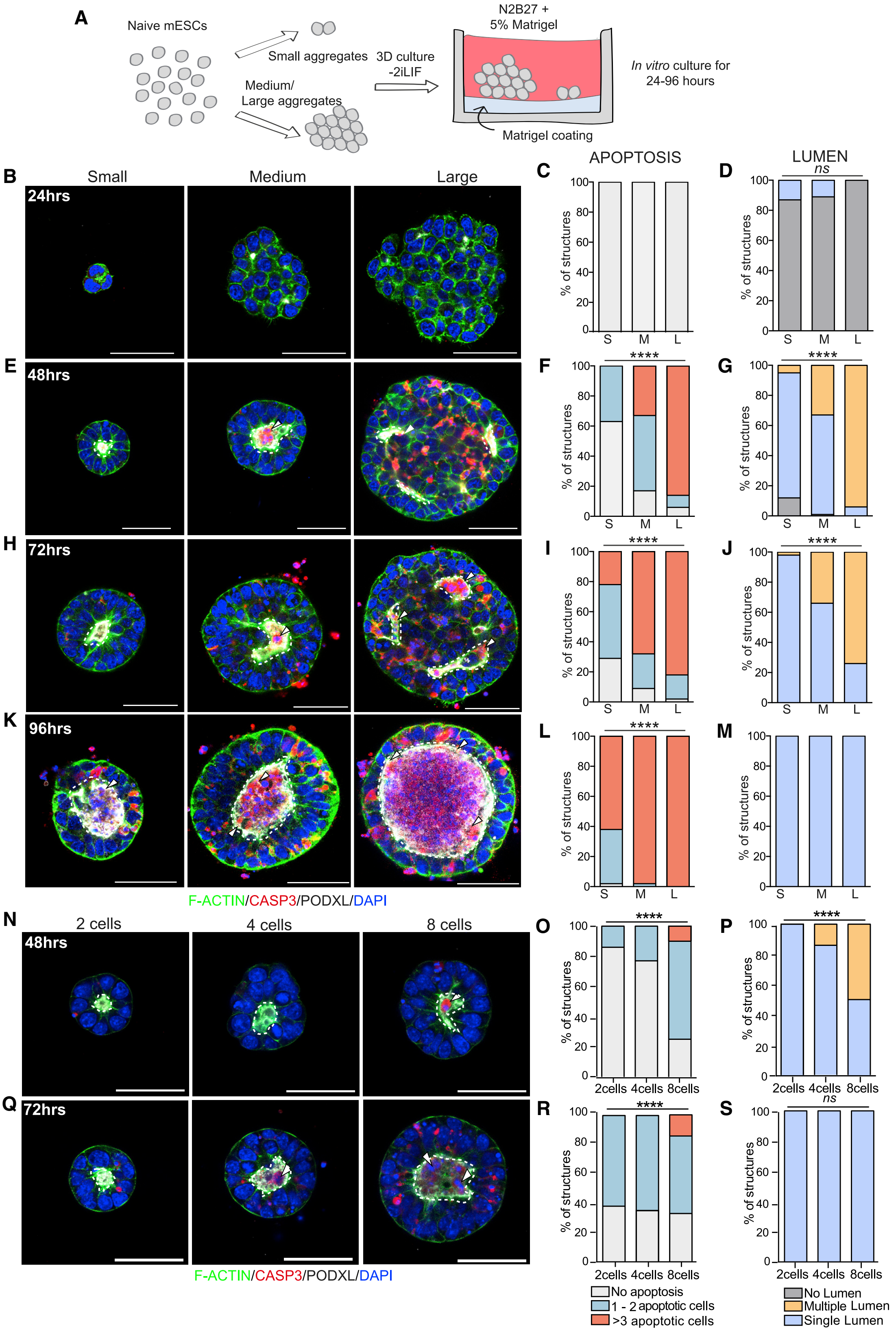

(legend on next page)

apoptosis (Figures 3N [arrowheads], 3O, 3Q [arrowheads], and 3R) and approximately half of them displayed a multi-lumen phenotype 48 h after cell seeding (Figure 3P). Importantly, multi-lumen aggregates had on average significantly more cells than aggregates with a single lumen (Figure S1Q), indicating that the mechanism of lumen formation is density dependent. By 72 h, all aggregates, irrespective of their initial size, displayed a single lumen (Figure 3S), in agreement with our results above (Figure 3M).

To identify the dynamics of lumen formation, we imaged this process following induction of the expression of a GFP-tagged PODOCALYXIN (GFP-PODXL). To monitor cell death in living samples, we added SYTOX to the medium (Roth et al., 1997). The analysis of our time-lapse movies confirmed that in large aggregates, the accumulation of PODOCALYXIN and multiple lumen formation initiated after 24 h in culture. However, opening of a single cavity was only achieved upon the death of inside cells, which took place between 72 and 96 h in culture (Figures S3F–S3H and Videos S1, S2, and S3). Taken together, these results suggest that in medium and large aggregates, small lumens initially emerge via hollowing, but their fusion into a single cavity relies on cell death.

Inhibition of Apoptosis Prevents Single Lumen Formation in Large ESC Aggregates

Since our results suggested that apoptosis might be involved in fusion of smaller cavities into a unified lumen, we next sought to test whether inhibiting the apoptotic pathway would affect the ability of large aggregates to form single lumens. With this aim, we used the pan-caspase inhibitor Z-VAD-FMK and cultured ESC aggregates of varied sizes in 3D as previously. We found that following Z-VAD-FMK treatment, small, medium, and large ESC aggregates showed fewer dead cells compared with control aggregates, even though apoptosis was not fully inhibited (Figure S4A–S4D). In agreement with this, we observed a delay in single lumen formation in large aggregates, although lumenogenesis was achieved by 96 h of culture (Figures S4E and S4F).

Because Z-VAD-FMK treatment was not sufficient to completely block apoptosis, we next prevented cell death by overexpressing the pro-survival gene *Bcl-2*, which suppresses apoptosis without affecting the ability of ESCs to self-renew or to differentiate (Yamane et al., 2005) (Figure S4G). We generated aggregates of BCL-2 overexpressing ESCs, classified them into different groups according to their size (Figures S4H–S4M), and evaluated their development over 96 h. Under these conditions apoptosis was drastically reduced, even though a proportion of aggregates had few cleaved CASPASE-3-positive cells (Figures 4B, 4E, and 4H). We observed that inhibition of apoptosis had no effect at 24 h (data not shown), consistent with our inability to detect apoptosis in wild-type ESC aggregates during the first day in culture (Figure 3C). By 48 h, small aggregates were unaffected by suppression of apoptosis and underwent lumenogenesis via hollowing (Figures 4A [left panel] and 4C). Large aggregates were also unaffected and preserved their typical multi-lumen morphology (Figures 4A [middle and right panels] and 4C), similar to apoptosis-proficient cells. This finding strengthens the notion that multiple lumen formation in medium and large aggregates results from hollowing and is independent of apoptosis. However, at later time points, single lumen formation was almost completely abolished by the suppression of apoptosis in large aggregates (Figures 4F–4I). In particular, over 60% and 90% of medium and large aggregates, respectively, never achieved single lumen formation, whereas small aggregates underwent lumenogenesis with an efficiency similar to that of control cells (Figure 4I). Together, our results demonstrate that multiple small lumens are formed independently of apoptosis in large ESC aggregates but that cell death is required for their fusion into a unified single cavity.

Figure 3. Cell Density Determines the Dynamics of Lumen Formation in Mouse ESCs

(A) Schematic representation of experimental procedure for the generation and culture of mouse ESC aggregates.

(B–S) Representative images of small, medium, and large mouse ESC aggregates at 24 (B), 48 (E), 72 (H), and 96 (K) h after removal of naive pluripotency factors. Percentage of mouse ESC aggregates with no apoptotic cells (light gray), 1–2 apoptotic cells (green), or more than 3 apoptotic cells (red) in the lumen at 24 (C), 48 (F), 72 (I), and 96 (L) h. Percentage of mouse ESC aggregates of different sizes showing no lumen (gray), single lumen (blue), or multiple lumens (yellow) at 24 (D), 48 (G), 72 (J), and 96 (M) h. Lumens are marked by PODOCALYXIN (PODXL). At 24 h, small aggregates n = 45, medium aggregates n = 44, large aggregates n = 44. At 48 h, small aggregates n = 51, medium aggregates n = 53, large aggregates n = 51. At 72 h, small aggregates n = 45, medium aggregates n = 47, large aggregates n = 45. At 96 h, small aggregates n = 47, medium aggregates n = 50, large aggregates n = 47. Three independent experiments. Representative images of 2-, 4-, and 8-cell aggregates at 48 (N) and 72 (Q) h after plating. Percentage of mouse ESC aggregates with no apoptotic cells (light gray), 1–2 apoptotic cells (green), or more than 3 apoptotic cells (red) in the lumen at 48 h (O) and 72 h (R). Percentage of mouse ESC aggregates showing single lumens (blue) or multiple lumens (yellow) at 48 h (P) and 72 h (S). At 48 h, 2-cell aggregates n = 28, 4-cell aggregates n = 22, 8-cell aggregates n = 20. At 72 h, 2-cell aggregates n = 21, 4-cell aggregates n = 18, 8-cell aggregates n = 30. Two independent experiments. Statistical analyses: χ^2 test. ****$p < 0.0001$; *ns*, not significant. Arrowheads indicate cleaved CASPASE-3 (CASP3)-positive cells, dotted lines indicate lumens. S, small; M, medium; L, large. Scale bars, 50 μm.

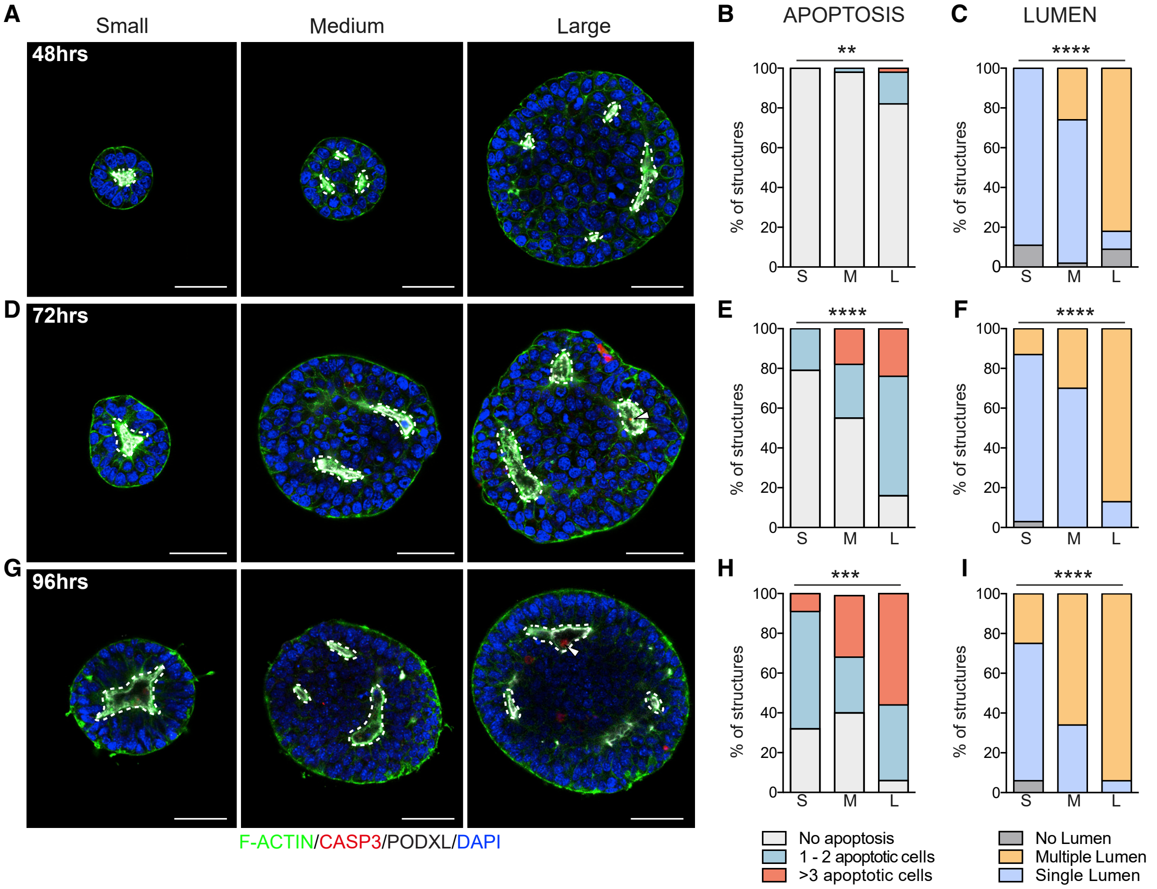

Figure 4. Inhibition of Apoptosis Prevents Single Lumen Formation in Large Mouse ESC Aggregates
Representative images of apoptosis-deficient BCL-2 overexpressing mouse ESC aggregates at 48 (A), 72 (D), and 96 (G) h after removal of naive pluripotency factors. Percentage of mouse ESC aggregates with no apoptotic cells (light gray), 1–2 apoptotic cells (green), or more than 3 apoptotic cells (red) in the lumen at 48 (B), 72 (E) and 96 (H) h. Percentage of mouse ESC aggregates of different sizes showing no lumen (gray), single lumens (blue), or multiple lumens (yellow) at 48 (C), 72 (F), and 96 (I) h. Lumens are marked by PODOCALYXIN (Podxl). At 48 h, small aggregates n = 45, medium aggregates n = 46, large aggregates n = 44. At 72 h, small aggregates n = 38, medium aggregates n = 40, large aggregates n = 38. At 96 h, small aggregates n = 32, medium aggregates n = 32, large aggregates n = 32. Three independent experiments. Statistical analyses: χ^2 test. ****$p < 0.0001$, ***$p < 0.001$, **$p < 0.01$. Arrowheads indicate cleaved CASPASE-3 (CASP3)-positive cells, dotted lines indicate lumens. S, small; M, medium; L, large. Scale bars, 50 μm.

Inhibition of Apoptosis Does Not Prevent Size Regulation but Inhibits Pro-amniotic Cavity Formation in Double Embryos *In Vivo*

We next wished to gain functional evidence of the role of apoptosis in the processes of size regulation and pro-amniotic cavity formation *in vivo*. To this end, we aggregated BCL-2-overexpressing cells labeled with H2B-GFP (H2B-GFP BCL-2 OE) with pre-implantation mouse embryos. To test the effect of apoptosis in enlarged embryos, we first generated both single and double embryos at the 8-cell stage, as previously, then aggregated them with either 3–4 or 8–10 H2B-GFP BCL-2 OE ECSs, respectively (Figure 5A). We found that at the blastocyst stage, double chimeric embryos showed an increased epiblast size when compared with single embryos (Figures 5B–5D). We transferred these single and double chimeric embryos to pseudo-pregnant mothers and recovered them at E5.5. We found that 3 of 12 single embryos and 2 of 17 double embryos were disorganized with gross morphological abnormalities and were therefore excluded from the analysis. In addition, 1 of 12 single embryos and 3 of 17 double embryos showed a low degree of chimerism in the epiblast (i.e., less than 40%) and were also excluded (Table S1). The remaining single and double embryos had high epiblast chimerism (Figure 5F) and double embryos were approximately 1.5 times bigger, suggesting that size regulation was not yet completed (Figure 5G). At this stage, the vast majority of double embryos (83%, n = 12) presented a multi-layered

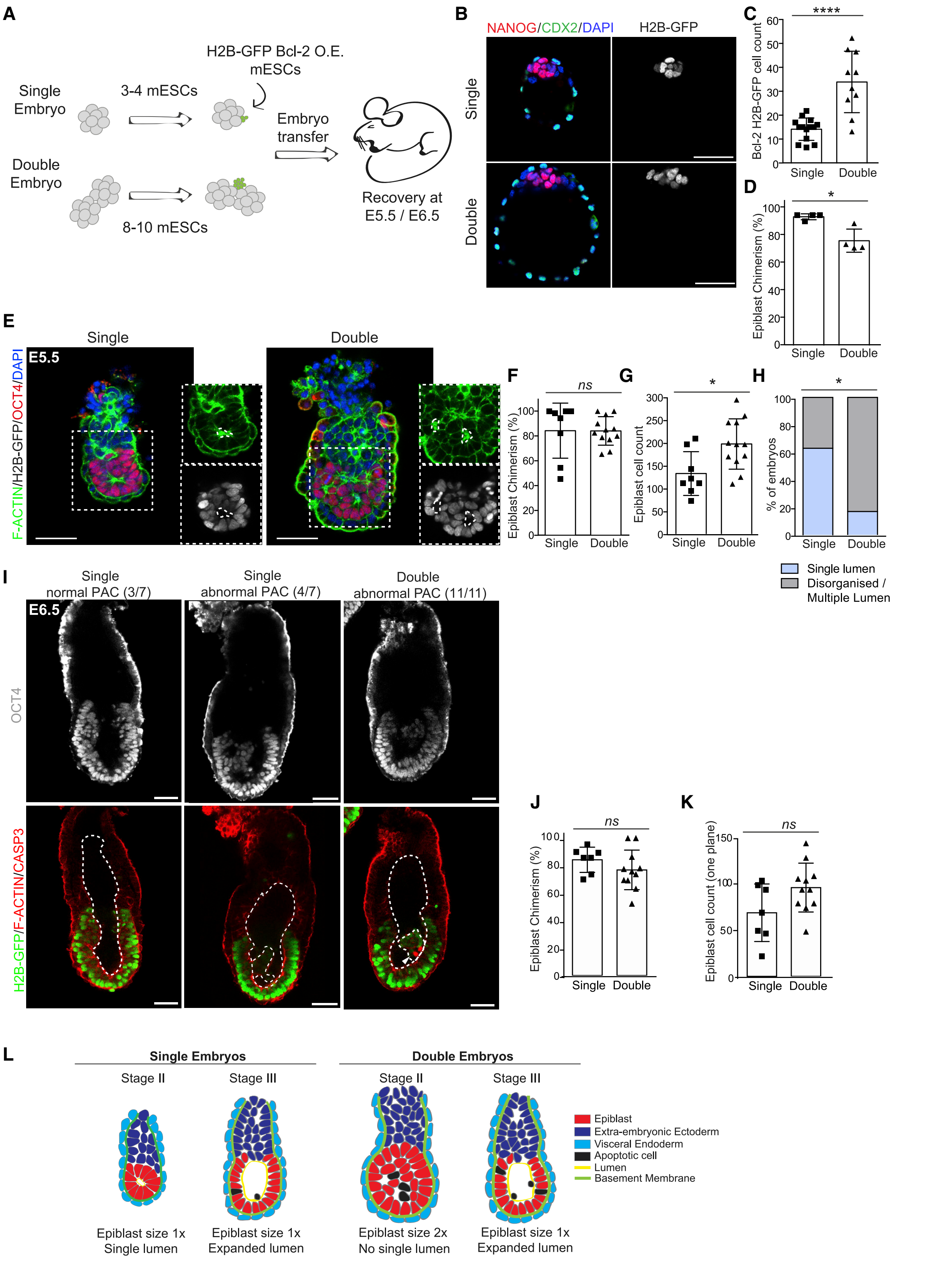

(legend on next page)

epiblast that did not form a single lumen (Figures 5E and 5H). In contrast, suppression of apoptosis did not impair epiblast epithelialization or lumen formation in single embryos (63%, n = 8) (Figures 5E and 5H; Table S1). These results indicate that apoptosis is required for single lumen formation in double but not normal-sized embryos.

It has been reported that regulation of embryo size is achieved by cell-cycle lengthening rather than apoptosis (Buehr and Mclaren, 1974; Lewis and Rossant, 1982). To functionally validate the effects of apoptosis inhibition on size regulation, we repeated the chimera experiment, as above, but recovered embryos at E6.5. In this case, 1 of 8 single embryos and 1 of 14 double embryos presented gross morphological abnormalities and therefore were excluded from the analysis (Table S2). In addition, 2 of 14 double embryos were also excluded as they were developmentally advanced (i.e., gastrulating). Evaluation of cell numbers and epiblast morphology revealed that in double embryos apoptosis-deficient epiblasts underwent size regulation but presented defects in pro-amniotic cavity formation, such as multi-lumen formation, specifically in double embryos (Figures 5I–5K and Table S2). Taken together, our findings demonstrate that cell death plays a marginal role in embryo downsizing but is essential for correct epiblast morphogenesis when the number of cells in the embryo is increased. We conclude, therefore, that size regulation and epiblast morphogenesis, albeit concomitant, have distinct molecular underpinnings.

DISCUSSION

Early mammalian embryos show remarkable plasticity—in other words, they are able to respond to external perturbations by altering the normal mechanisms of development and therefore ensuring developmental progression. Paradigmatic examples of this plasticity are the ability of cells to acquire different fates following splitting of the embryo into smaller parts, and the regulation of embryo size that takes place when multiple embryos are aggregated together (Rands, 1986a; Power and Tam, 1993, Buehr and Mclaren, 1974). In agreement with previous reports (Lewis and Rossant, 1982; Saiz et al., 2016), our data show that regulation of embryo size takes place only when an embryo starts to grow after implantation and can sense alterations in cell numbers. It has been shown that the regulation of embryo size happens through the modulation of the cell-cycle machinery (Buehr and Mclaren, 1974; Lewis and Rossant, 1982). However, whether, and if so how, embryo size affects the timing and mechanisms of morphogenesis remains unknown.

To address this question, we have used double-sized mouse embryos as a model system and focused on the morphogenesis of the epiblast during the first days of post-implantation development. Applying confocal microscopy and different criteria to stage embryo morphogenesis, we have demonstrated that pro-amniotic cavity formation is delayed in double embryos compared with controls. Previous studies analyzed bright-field images of *in vivo* developing single and double embryos. However, while some suggested that pro-amniotic cavity formation precedes size regulation (Lewis and Rossant, 1982), others proposed that size regulation takes place before pro-amniotic cavity formation (Buehr and Mclaren, 1974). Our findings resolve this discrepancy and demonstrate that size regulation in enlarged embryos and epiblast morphogenesis happen concomitantly. We conclude, therefore, that

Figure 5. Inhibition of Apoptosis Prevents Lumenogenesis in Double Embryos *In Vivo*
(A) Schematic representation of the experimental procedure for the aggregation of embryo with H2B-GFP BCL-2 O.E. mouse ESCs (green).
(B) Representative images of single and double embryos after aggregation of H2B-GFP BCL-2 O.E. ESCs cultured *in vitro* to the blastocyst stage.
(C) Cell counts of H2B-GFP BCL-2 O.E. cells at the blastocyst stage.
(D) Contribution of H2B-GFP BCL-2 O.E. cells to the epiblast at the blastocyst stage.
(E) Representative images of single and double embryos with apoptosis-deficient epiblast at E5.5.
(F) Contribution of H2B-GFP BCL-2 O.E mouse ESCs to the epiblast of single and double embryos at E5.5.
(G) Epiblast cell counts (OCT4-positive cells) at E5.5 in single and double embryos upon aggregation of H2B-GFP BCL-2 O.E. cells.
(H) Percentage of single and double embryos with apoptosis-deficient epiblast showing disorganized/multiple lumens (gray) or single lumens (blue) at E5.5.
(I) Representative images of single embryos and double embryos with apoptosis-deficient epiblast at E6.5.
(J) Contribution of H2B-GFP BCL-2 O.E ESCs to the epiblast of single and double embryos at E6.5.
(K) Epiblast cell counts (OCT4-positive cells) at E6.5 of single and double embryos upon aggregation of H2B-GFP BCL-2 O.E. cells.
(L) Working model describing the morphological events in double embryo early post-implantation development.
In (C), single embryos n = 14, double embryos n = 10. In D, single embryos n = 4, double embryos n = 4. Two independent experiments. At E5.5, single embryos n = 8, double embryos n = 12. Four independent experiments. At E6.5, single embryos n = 7, double embryos n = 11. Two independent experiments. In (C), (D), (F), (G), (J), and (K), statistical analyses: Student's t test. ****$p < 0.0001$, *$p < 0.05$; *ns*, not significant. In (H), statistical analyses: χ^2 test. *$p < 0.05$. Bar charts display mean ± SD. Dotted lines indicate lumens and squares mark magnified regions. Arrowheads indicate cleaved CASPASE-3 (CASP3)-positive cells in the pro-amniotic cavity (PAC). Scale bars, 50 μm.

embryo size modulates the timing of morphogenesis (Figure 5L). Interestingly, although size regulation does not take place until early organogenesis in embryos with a reduced number of cells (i.e., half embryos), the timing of morphogenesis (specifically primitive streak formation) is also delayed (Power and Tam, 1993; Rands, 1986b). Future studies should allow determination of the dynamics of pro-amniotic cavity formation in half embryos, but our results indicate that embryo size modulates the morphogenetic tempo.

We next explored the mechanisms of pro-amniotic cavity formation in double embryos. It has been shown that when cell density is increased in *in vitro* 3D cultures of MDCK cells, lumens form not by hollowing but by apoptosis-induced cavitation (Martin-Belmonte et al., 2008). Therefore, we wished to establish whether this mechanism is physiologically relevant in mouse embryos. By quantifying the number of cleaved CASPASE-3-positive cells at different developmental stages, we detected an increase in the levels of apoptosis in double embryos compared with controls, specifically at the time of pro-amniotic cavity formation. We found that the apoptotic index in the epiblast of double embryos was approximately 5%, in agreement with previous estimates (Lewis and Rossant, 1982). We observed that cells undergoing apoptosis lack contact with the basement membrane. This result is in agreement with studies showing that survival of epithelial cells depends on direct contact with the basement membrane (Frisch and Francis, 1994), which in turn activates pro-survival pathways, such as PI3K and MEK, downstream of integrin signaling (Debnath et al., 2002; Giannoni et al., 2008).

To dissect the mechanism of lumenogenesis in enlarged epiblasts, we developed an assay that allowed us to assess how variations in size affect lumen formation in ESCs. We generated ESC aggregates of varying sizes and cultured them in Matrigel as the *in vitro* source of extracellular matrix. This revealed that in medium and large aggregates, outer cells polarize and form small lumens by hollowing while inner cells not in contact with the surrounding extracellular matrix undergo apoptosis. This leads to the fusion of small lumens into a single unified cavity. Importantly, when ESC aggregates are grown in agarose, outer cells undergo apoptosis. Therefore, our results show that survival of both epiblast cells in embryos and ESCs *in vitro* is mediated by anchorage to the underlying basement membrane. Our findings could also be relevant in other *in vivo* and *in vitro* contexts of epithelial tissue formation and lumenogenesis. It is tempting to speculate that the mechanisms of lumen formation in adult stem cell organoids are dependent on cellular density. Similarly, the interactions between adult epithelial cells and basement membrane components may regulate cell survival and tissue remodeling during regeneration.

In the embryo, deposition of basement membrane components is first observed in the extra-embryonic lineages by the blastocyst stage (Boroviak et al., 2015; Li et al., 2003). Shortly after implantation, the basement membrane is required for the establishment of apicobasal polarity and lumenogenesis in the epiblast (Bedzhov and Zernicka-Goetz, 2014) and the extra-embryonic ectoderm (Christodoulou et al., 2018). However, lack of contact with the basement membrane does not result in activation of apoptosis in the extra-embryonic ectoderm compartment, which suggests that specific tissues respond differently to a given morphogenetic cue. Future studies will be needed to determine how increased cell numbers affect the mechanism of lumenogenesis in the extra-embryonic ectoderm compartment.

Previous reports have shown that a subset of epiblast cells undergoes apoptosis during early post-implantation development as a result of cell competition (Claveria et al., 2013; Sancho et al., 2013). Cells with low levels of Myc and mTOR, and high levels of p53 and ERK phosphorylation are selectively eliminated by programmed cell death (Bowling et al., 2018; Diaz-Diaz et al., 2017). Whether elimination of cells with lower fitness contributes to lumen opening in double embryos remains to be explored.

To determine the consequences of apoptosis inhibition for pro-amniotic cavity formation, we overexpressed the anti-apoptotic factor BCL2 in ESC cells and used them to generate 3D ESC aggregates of different size *in vitro* and single- and double-size chimeric embryos *in vivo*. These experiments revealed that apoptosis is required for the fusion of small cavities into a single unified pro-amniotic cavity specifically in double embryos. Therefore, while in normal-sized embryos pro-amniotic cavity formation is triggered by polarization and exocytosis (Bedzhov and Zernicka-Goetz, 2014; Shahbazi et al., 2017), an increase in epiblast cell numbers activates an apoptosis-dependent mechanism of cavity formation. Remarkably, embryos harboring an apoptosis-resistant epiblast were still able to regulate their size. These results indicate that albeit concomitant, pro-amniotic cavity formation and size regulation have different molecular underpinnings. Given that approximately 34% of double embryos give rise to live pups (Mintz, 1965), we can conclude that at least in a proportion of embryos morphogenesis correction via apoptosis and size regulation are successfully achieved.

In conclusion, our study shows that embryo size determines the mechanism and timing of morphogenesis of the embryonic tissue. Understanding how this is coordinated with the morphogenesis and size regulation of the extra-embryonic tissues will not only provide a complete picture of the mechanisms behind mammalian embryo plasticity but will also be potentially relevant for unraveling the mechanisms of organ and organism size control.

EXPERIMENTAL PROCEDURES

Embryo Recovery and Culture

Mice were bred in the Animal House of the Gurdon Institute in accordance with national and international guidelines. Experiments were approved under the Animals (Scientific Procedures) Act 1986 Amendment Regulations 2012 following ethical review by the University of Cambridge Animal Welfare and Ethical Review Body. For embryo production, F1 (C57Bl/6 × CBA) females were superovulated by injection with 7.5 IU of pregnant mare's serum gonadotropin (Intervet) followed by injection with 7.5 IU of human chorionic gonadotropin (Intervet) 48 h later and mating with F1 males. Pre-implantation embryos were collected at E2.5 from the oviducts and uteri in drops of M2 medium. Double embryos were generated by removal of *zona pellucida* with brief incubation in acidic Tyrode's solution (T1788, Sigma-Aldrich) and aggregation of two E2.5 embryos in drops of KSOM. Single embryos also underwent a brief incubation in acidic Tyrode's solution to remove the *zona pellucida*. Single and double embryos were cultured in KSOM (MR-020P-5F, Millipore) covered by mineral oil at 37°C in 5% CO_2. For transfer experiments, early blastocysts (equivalent to E3.5) were transferred into F1 foster females at E0.5 or E2.5 of pseudo-pregnancy and recovered at E5.5. All embryos were recovered at the same time (10 a.m.), but due to their heterogeneous development we were able to classify them into different morphological stages. Post-implantation embryos were dissected out from the decidua and immediately fixed.

In Vitro Culture of Isolated ICMs through Post-implantation Stages

To isolate ICMs, we subjected late blastocysts to immunosurgery (Solter and Knowles, 1975). In brief, *in vitro* cultured single and double embryos were incubated with anti-mouse whole serum (M5774-2ML, Sigma-Aldrich) and sera complement guinea pig (S1639-5ML, Sigma-Aldrich) diluted to 20% in M2 medium for 45 min each. After elimination of trophectoderm cells by pipetting with a narrow glass capillary in M2 medium, ICMs were seeded in individual hanging drops of pre-warmed IVC-1 medium (Bedzhov et al., 2014) (M11-25, Cell Guidance Systems) and cultured for 24, 48, and 72 h at 37°C in 5% CO_2 (Figure 2A).

Embryo/Mouse ESC Aggregation Chimeras

Embryo/mouse ESC aggregation was carried out with pre-compaction 8-cell stage embryos devoid of *zona pellucida*. For the double embryo group, two 8-cell stage embryos were fused as described above and then aggregated to a clump containing 8–10 H2B-GFP BCL-2 O.E mouse ESCs. For single embryos, we used 3–4 H2B-GFP BCL-2 O.E mouse ESCs. Before aggregation, mouse ESCs were prepared as follows. Cells were first incubated with trypsin-EDTA for 5 min to obtain single cells and plated in non-adherent dishes for 1 h in N2B27+2iLIF to allow cells to coalesce and form small aggregates. Thereafter, aggregates of the appropriate size were manually picked with a narrow glass capillary and deposited onto single or double embryos in drops of KSOM medium. Embryo/mouse ESC aggregates were cultured in KSOM at 37°C in 5% CO_2, for 48 h for cell count at the blastocyst stage or for 24 h for embryo transfer experiments.

Generation of Mouse ESC Aggregates

For generation of mouse ESC aggregates of different sizes, 100,000, 50,000, and 5,000 single cells were seeded in individual wells in non-adherent 24-well plate (662102, Greiner Bio-One) in N2B27 supplemented with 2iLIF. On the following day all aggregates were collected, mixed, and washed with PBS to remove any remaining 2iLIF. They were then plated in 8-well ibiTreat (IB-80826, Ibidi) dishes previously coated with 40 μL of growth factor reduced Matrigel (356230, BD Biosciences) in N2B27. Upon attachment to the gel, the medium was replaced with N2B27 containing 5% Matrigel, and aggregates were cultured for 24–96 h with media change every 48 h. To generate mouse ESC aggregates with defined size, we manually picked ESC aggregates containing two, four, or eight cells and plated them using the same method as described above. For apoptosis inhibition, Z-VAD-FMK (ALX-260-020-M001, Enzo Life Sciences) was used at 20 μM. For plating of mouse ESC aggregates in agarose, 1% low-melting-point agarose (16520050, Thermo Fisher Scientific) was dissolved in PBS. Cell aggregates were resuspended and plated in a single drop of agarose in 8-well ibiTreat dishes covered with N2B27 supplemented with naive pluripotency factors (2iLIF) or N2B27 alone.

Data and Code Availability

All relevant data are available from the corresponding authors upon reasonable request.

SUPPLEMENTAL INFORMATION

Supplemental Information can be found online at https://doi.org/10.1016/j.stemcr.2020.09.004.

AUTHOR CONTRIBUTIONS

L.C.O., V.S.R., M.N.S., and F.A. designed and performed the experiments with help from C.K. W.M. performed embryo transfer experiments. L.C.O., V.S.R., and M.N.S. analyzed the data. L.C.O., V.S.R., M.N.S., and M.Z.-G. wrote the paper. H.M.-S. supported the work of V.S.R. M.N.S. and M.Z.-G. supervised the study. M.Z.-G. conceived the project.

ACKNOWLEDGMENTS

We acknowledge Neophytos Christodoulou, Bailey Weatherbee, and David Glover for discussion and feedback on the manuscript. L.C.O. was funded by a PhD fellowship from the Centre for Trophoblast Research, University of Cambridge. V.S.R. was supported by the CAPES (88881.132023/2016-01). F.A. was funded by an EMBO long-term fellowship. Work in the M.N.S. laboratory is funded by the European Molecular Laboratory (EMBO—Advanced EMBO fellowship) and the Medical Research Council (MC_UP_1201/24). Work in the M.Z.-G. laboratory is funded by a Wellcome Trust grant (207415/Z/17/Z), an ERC grant (669198), Open Philanthropy, Curci, and Weston Havens Foundations at Caltech.

Received: June 4, 2020
Revised: September 1, 2020
Accepted: September 8, 2020
Published: October 8, 2020

REFERENCES

Banfalvi, G. (2017). Methods to detect apoptotic cell death. Apoptosis *22*, 306–323.

Bedzhov, I., Leung, C.Y., Bialecka, M., and Zernicka-Goetz, M. (2014). In vitro culture of mouse blastocysts beyond the implantation stages. Nat. Protoc. *9*, 2732–2739.

Bedzhov, I., and Zernicka-Goetz, M. (2014). Self-Organizing properties of mouse pluripotent cells initiate morphogenesis upon implantation. Cell *156*, 1032–1044.

Boroviak, T., Loos, R., Lombard, P., Okahara, J., Behr, R., Sasaki, E., Nichols, J., Smith, A., and Bertone, P. (2015). Lineage-specific profiling delineates the emergence and progression of naive pluripotency in mammalian embryogenesis. Dev. Cell *35*, 366–382.

Bowling, S., Di Gregorio, A., Sancho, M., Pozzi, S., Aarts, M., Signore, M.D., Martinez-Barbera, J.P., Gil, J., and Rodriguez, T.A. (2018). P53 and mTOR signalling determine fitness selection through cell competition during early mouse embryonic development. Nat. Commun. *9*, 1763.

Buehr, M., and Mclaren, A. (1974). Size regulation in chimaeric mouse embryos. J. Embryol. Exp. Morphol. *31*, 229–234.

Christodoulou, N., Kyprianou, C., Weberling, A., Wang, R., Cui, G., Peng, G., Jing, N., and Zernicka-Goetz, M. (2018). Sequential formation and resolution of multiple rosettes drive embryo remodelling after implantation. Nat. Cell Biol. *20*, 1278–1289.

Claveria, C., Giovinazzo, G., Sierra, R., and Torres, M. (2013). Myc-driven endogenous cell competition in the early mammalian embryo. Nature *500*, 39–44.

Coucouvanis, E., and Martin, G.R. (1995). Signals for death and survival: a two-step mechanism for cavitation in the vertebrate embryo. Cell *83*, 279–287.

Datta, A., Bryant, D.M., and Mostov, K.E. (2011). Molecular regulation of lumen morphogenesis. Curr. Biol. *21*, R126–R136.

Debnath, J., Mills, K.R., Collins, N.L., Reginato, M.J., Muthuswamy, S.K., and Brugge, J.S. (2002). The role of apoptosis in creating and maintaining luminal space within normal and oncogene-expressing mammary acini. Cell *111*, 29–40.

Diaz-Diaz, C., Fernandez de Manuel, L., Jimenez-Carretero, D., Montoya, M.C., Claveria, C., and Torres, M. (2017). Pluripotency surveillance by Myc-driven competitive elimination of differentiating cells. Dev. Cell *42*, 585–599 e4.

Frisch, S.M., and Francis, H. (1994). Disruption of epithelial cell-matrix interactions induces apoptosis. J. Cell Biol *124*, 619–626.

Giannoni, E., Buricchi, F., Grimaldi, G., Parri, M., Cialdai, F., Taddei, M.L., Raugei, G., Ramponi, G., and Chiarugi, P. (2008). Redox regulation of anoikis: reactive oxygen species as essential mediators of cell survival. Cell Death Differ. *15*, 867–878.

Lawrence, P.A., and Levine, M. (2006). Mosaic and regulative development: two faces of one coin. Curr. Biol. *16*, R236–R239.

Lewis, N.E., and Rossant, J. (1982). Mechanism of size regulation in mouse embryo aggregates. J. Embryol. Exp. Morphol. *72*, 169–181.

Li, S., Edgar, D., Fassler, R., Wadsworth, W., and Yurchenco, P.D. (2003). The role of laminin in embryonic cell polarization and tissue organization. Dev. Cell *4*, 613–624.

Martin-Belmonte, F., Yu, W., Rodriguez-Fraticelli, A.E., Ewald, A.J., Werb, Z., Alonso, M.A., and Mostov, K. (2008). Cell-polarity dynamics controls the mechanism of lumen formation in epithelial morphogenesis. Curr. Biol. *18*, 507–513.

Mintz, B. (1965). Genetic mosaicism in adult mice of quadriparental lineage. Science *148*, 1232–1233.

Neagu, A., van Genderen, E., Escudero, I., Verwegen, L., Kurek, D., Lehmann, J., Stel, J., Dirks, R.A.M., van Mierlo, G., Maas, A., et al. (2020). In vitro capture and characterization of embryonic rosette-stage pluripotency between naive and primed states. Nat. Cell Biol. *22*, 534–545.

Power, M.A., and Tam, P.P. (1993). Onset of gastrulation, morphogenesis and somitogenesis in mouse embryos displaying compensatory growth. Anat. Embryol. (Berl) *187*, 493–504.

Rands, G.F. (1986a). Size regulation in the mouse embryo. I. The development of quadruple aggregates. J. Embryol. Exp. Morphol. *94*, 139–148.

Rands, G.F. (1986b). Size regulation in the mouse embryo. II. The development of half embryos. J. Embryol. Exp. Morphol. *98*, 209–217.

Roth, B.L., Poot, M., Yue, S.T., and Millard, P.J. (1997). Bacterial viability and antibiotic susceptibility testing with SYTOX green nucleic acid stain. Appl. Environ. Microbiol. *63*, 2421–2431.

Saiz, N., Williams, K.M., Seshan, V.E., and Hadjantonakis, A.K. (2016). Asynchronous fate decisions by single cells collectively ensure consistent lineage composition in the mouse blastocyst. Nat. Commun. *7*, 13463.

Sancho, M., di-Gregorio, A., George, N., Pozzi, S., Sanchez, J.M., Pernaute, B., and Rodriguez, T.A. (2013). Competitive interactions eliminate unfit embryonic stem cells at the onset of differentiation. Dev. Cell *26*, 19–30.

Shahbazi, M.N., Jedrusik, A., Vuoristo, S., Recher, G., Hupalowska, A., Bolton, V., Fogarty, N.M., Campbell, A., Devito, L.G., Ilic, D., et al. (2016). Self-organization of the human embryo in the absence of maternal tissues. Nat. Cell Biol. *18*, 700–708.

Shahbazi, M.N., Scialdone, A., Skorupska, N., Weberling, A., Recher, G., Zhu, M., Jedrusik, A., Devito, L.G., Noli, L., Macaulay, I.C., et al. (2017). Pluripotent state transitions coordinate morphogenesis in mouse and human embryos. Nature *552*, 239–243.

Shahbazi, M.N., and Zernicka-Goetz, M. (2018). Deconstructing and reconstructing the mouse and human early embryo. Nat. Cell Biol. *20*, 878–887.

Sheng, G. (2015). Epiblast morphogenesis before gastrulation. Dev. Biol. *401*, 17–24.

Slee, E.A., Harte, M.T., Kluck, R.M., Wolf, B.B., Casiano, C.A., Newmeyer, D.D., Wang, H.G., Reed, J.C., Nicholson, D.W., Alnemri, E.S., et al. (1999). Ordering the cytochrome c-initiated caspase cascade: hierarchical activation of caspases-2, -3, -6, -7, -8,

and -10 in a caspase-9-dependent manner. J. Cell Biol. *144*, 281–292.

Solter, D., and Knowles, B.B. (1975). Immunosurgery of mouse blastocyst. Proc. Natl. Acad. Sci. U S A *72*, 5099–5102.

Tarkowski, A.K. (1961). Mouse chimaeras developed from fused eggs. Nature *190*, 857–860.

Tarkowski, A.K., and Wroblewska, J. (1967). Development of blastomeres of mouse eggs isolated at the 4- and 8-cell stage. J. Embryol. Exp. Morphol. *18*, 155–180.

Yamane, T., Dylla, S.J., Muijtjens, M., and Weissman, I.L. (2005). Enforced Bcl-2 expression overrides serum and feeder cell requirements for mouse embryonic stem cell self-renewal. Proc. Natl. Acad. Sci. U S A *102*, 3312–3317.

Stem Cell Reports, Volume *16*

Supplemental Information

Embryo size regulates the timing and mechanism of pluripotent tissue morphogenesis

Lorenzo C. Orietti, Viviane Souza Rosa, Francesco Antonica, Christos Kyprianou, William Mansfield, Henrique Marques-Souza, Marta N. Shahbazi, and Magdalena Zernicka-Goetz

SUPPLEMENTARY INFORMATION

Embryo size regulates the timing and mechanism of pluripotent tissue morphogenesis

Lorenzo C. Orietti[1,2#], Viviane Souza Rosa[1,3#], Francesco Antonica[1,4], Christos Kyprianou[1], William Mansfield[5], Henrique Marques-Souza[3], Marta N. Shahbazi[1,6*], Magdalena Zernicka-Goetz[1,2,7*]

SUPPLEMENTARY FIGURE LEGENDS

FIGURE S1: Further characterization of single and double embryos and ESC aggregates. Related to Figure 1 and Figure 3.

Representative images of single and double embryos recovered at the 8-cell stage and cultured *in vitro* to the blastocyst stage (**A**). Cell counts in single and double blastocysts (**B**)**.** Representative image of double embryo at stage **III** showing a multiple lumen phenotype (**C**). Square denote magnified region and arrows indicate multi-lumens in **C.** Distribution and size of ESC aggregates at 24 (**D, E**), 48 (**F, G**), 72 (**H, I**) and 96 (**J, K**) hours after removal of naïve pluripotency factors. Representative image of a multi-layered ESC aggregate at 48 hours stained for F-ACTIN and GM130 (**L**). Calculation of the cell aspect ratio in outer and inner cells in ESC aggregates (**M**). Dotted lines mark cell height and continuous lines mark cell width. Squares denote magnified regions in **L** and arrows indicate GM130. Schematic representation illustrating Golgi localization in outer polarized and inner unpolarized cells (**N**). Number of cells of 2, 4 and 8 cell aggregates 48 (**O**) and 72 (**P**) hours after plating. Number of cells of 8 cells aggregates with single or multiple lumen at 48 hours (**Q**). In panel L Outer cells n=54, inner cells n=57 from 5 aggregates. In panel B single embryos n=16, double embryos n=5. In panels D-K and O-P, number of aggregates is indicated in Figure 3. In panel Q, aggregates with single lumen, n=10 and aggregates with multiple lumen, n=10. Statistical analyses: in panels B, M and Q, Student's *t*-test. ****p<0.0001, **p<0.01. In panels O and P, one-way ANOVA. ****p<0.0001. Dot plots display mean ± S.D. Scale bar 50 μm.

FIGURE S2: The rate of apoptosis increases upon exit from naïve pluripotency. Related to Figure 3.

Representative images of REX1::GFPd2 ESC aggregates cultured in basal medium alone (-2iLIF) (**A**, **B**) or basal medium supplemented with naïve pluripotency factors (+2iLIF) (**C**, **D**) for 72 or 96 hours. Percentage of ESC aggregates with no apoptotic cells (light grey), 1-2 apoptotic cells (green) or more than 3 apoptotic cells (red) in the aggregate at 72 (**E**) and 96 (**F**) hours. Percentage of ESC aggregates of different sizes showing no lumen (grey) or single and multiple lumens (blue) at 72 (**G**) and 96 (**H**) hours. At 72 hours, +2iLIF n=19, -2iLIF n=25. At 96 hours +2iLIF n=16, -2iLIF n=29. 1 independent experiment. Statistical analyses: χ^2 test. ***p<0.001, **p<0.01, *p<0.05, *ns*=not significant. Arrowheads indicate cleaved CASPASE-3 (CASP3) positive cells, dotted lines indicate lumens. Scale bars 50 μm.

FIGURE S3: Lack of basement membrane contact triggers apoptosis of ESCs cultured as aggregates. Related to Figure 3.

The basement membrane provides a survival signal for outside cells in ESC aggregates: Representative images of ESC aggregates cultured in matrigel (control) or in agarose for 24 (**A**), 48 (**B**), or 72 (**C**) hours after removal of naïve pluripotency factors. Arrowheads indicate cleaved CASPASE-3 (CASP3) positive cells, dotted lines indicate lumens. 1 independent experiment. Scale bars 50 μm. Representative images of ESC aggregates cultured in agarose with basal medium supplemented with naïve

pluripotency factors (+2iLIF) or basal medium alone (-2iLIF) (**D**) for 72 hours. Apoptotic index in ESC aggregates cultured in agarose with basal medium +2iLIF or -2iLIF (**E**). +2iLIF n=32, -2iLIF n=31. 3 independent experiments. Scale bars 30 μm. Statistical analyses: Mann Whitney. ****$p<0.0001$. Arrowheads indicate cleaved CASPASE-3 (CASP3) positive cells. Bar charts display mean ± SEM. Snapshots of representative GFP-PODXL (grey) overexpressing ESC small (**F**), medium (**G**) and large (**H**) aggregates cultured in the presence of SYTOX (green) and subjected to live imaging for 96 hours. Arrowheads indicate increased SYTOX signal, dotted lines indicate lumens. Scale bars 50 μm.

FIGURE S4: Chemical inhibition of apoptosis delays but does not prevent single lumen formation in large ESC aggregates. Related to Figure 4.

Representative images of ESC aggregates cultured in DMSO or with the apoptosis inhibitor Z-VAD-FMK (Z-VAD) for 72 (**A**) or 96 (**B**) hours after removal of naïve pluripotency factors. Percentage of ESC aggregates with no apoptotic cells (light grey), 1-3 apoptotic cells (green) or more than 3 apoptotic cells (red) in the lumen at 72 (**C**) and 96 (**D**) hours. Percentage of ESC aggregates of different sizes with no lumen (grey), single lumens (blue) or multiple lumens (yellow) at 72 (**E**) and 96 (**F**) hours. At 72 hours, DMSO n=21, Z-VAD n=28. At 96 hours DMSO n=36, Z-VAD n=37. 2 independent experiments. Statistical analyses: χ^2 test. ****$p<0.0001$, ***$p<0.001$, **$p<0.01$, *$p<0.05$, *ns* =not significant. Arrowheads indicate cleaved CASPASE-3 (CASP3) positive cells, dotted lines indicate lumens. Scale bars 50 μm. Classification of BCL-2 overexpressing ESC aggregates into different bins based on size**:** Representative images of control and Bcl-2 overexpressing ESC stained for OCT4 (red) and BCL-2 (grey) (**G**). Distribution and size of apoptosis deficient Bcl-2 overexpressing ESC aggregates at 48 (**H, I**), 72 (**J**, **K**) and 96 (**L**, **M**) hours after removal of naïve pluripotency factors. In panels H-M, number of aggregates is indicated in Figure 4. Dot plots display mean ± S.D. Scale bars 50 μm.

SUPPLEMENTARY TABLES

Table S1: Exclusion criteria for embryos with apoptosis-deficient epiblast at E5.5. Related to Figure 5.

E5.5	Disorganised	Low Chimerism	Single lumen	Multiple lumen	Disorganised EPI*
**Single**	3/12	1/12	5/8	2/8	1/8
**Double**	2/17	3/17	2/12	5/12	5/12

*EPI: epiblast.

Table S2: Exclusion criteria for embryos with apoptosis-deficient epiblast at E6.5. Related to Figure 5.

E6.5	Disorganised	Gastrulating	Low Chimerism	Normal PAC*	Abnormal PAC
**Single**	1/8	0/8	0/8	3/7	4/7
**Double**	1/14	2/14	0/14	0/11	11/11

*PAC: pro-amniotic cavity.

SUPPLEMENTARY MOVIES

MOVIE S1

Representative small ESC aggregate imaged from 24 to 96 hours after removal of naïve pluripotency factors. GFP–PODXL (grey), SYTOX (green). Scale bar 50 μm.

MOVIE S2

Representative medium ESC aggregate imaged from 24 to 96 hours after removal of naïve pluripotency factors. GFP–PODXL (grey), SYTOX (green). Scale bar 50 μm.

MOVIE S3

Representative large ESC aggregate imaged from 24 to 96 hours after removal of naïve pluripotency factors. GFP–PODXL (grey), SYTOX (green). Scale bar 50 μm.

SUPPLEMENTARY EXPERIMENTAL PROCEDURES

Mouse ESC culture

Mouse ESCs were cultured in gelatin coated plates in Fc or N2B27 containing the naïve pluripotency factors 2iLIF (1 µM MEK inhibitor PD0325901 (Stem Cell Institute), 3 µM GSK3 inhibitor CHIR99021 (Stem Cell Institute) and 10 ng ml^{-1} LIF (Stem Cell Institute)) at 37 °C, 5% CO_2, 21% O_2. Fc medium composition was DMEM (41966, Thermo Fisher Scientific), 15% fetal bovine serum (Stem Cell Institute), penicillin–streptomycin (15140122, Thermo Fisher Scientific), GlutaMAX (35050061, Thermo Fisher Scientific), MEM non-essential amino acids (11140035, Thermo Fisher Scientific), sodium pyruvate (11360070, Thermo Fisher Scientific) and 100 µM β-mercaptoethanol (31350-010, Thermo Fisher Scientific). N2B27 was prepared with a 1:1 mix of DMEM F12 (21331-020, Thermo Fisher Scientific) and neurobasal A (10888-022, Thermo Fisher Scientific) plus 1% v/v B27 (10889-038, Thermo Fisher Scientific), 0.5% v/v N2 (homemade), 100 µM β-mercaptoethanol (31350-010, Thermo Fisher Scientific), penicillin–streptomycin (15140122, Thermo Fisher Scientific) and GlutaMAX (35050061, Thermo Fisher Scientific). N2 supplement composition was DMEM F12 medium (21331-020, Thermo Fisher Scientific), 2.5 mg ml^{-1} insulin (I9287, Sigma-Aldrich), 10 mg ml^{-1} Apo-transferrin (T1147, Sigma-Aldrich), 0.75% bovine albumin fraction V (15260037, Thermo Fisher Scientific), 20 µg ml^{-1} progesterone (p8783, Sigma-Aldrich), 1.6 mg ml^{-1} putrescine dihydrochloride (P5780, Sigma-Aldrich) and 6 µg ml^{-1} sodium selenite (S5261, Sigma-Aldrich). Cells were passaged with trypsin-EDTA (25300054, Thermo Fisher Scientific) and routinely tested for contamination by mycoplasma.

E14 wild-type mouse ESCs were derived as previously described (Shahbazi *et al.*, 2017). Briefly, 8-cell stage 129aa mouse embryos were collected from the oviducts of pregnant females and cultured *in vitro* for 24 hours in KSOM supplemented with 2i, followed by 48 hours culture in N2B27 supplemented with 2iLIF. The resulting expanded and hatched blastocysts were plated in individual wells of a 96 well plate with mitomycin-C (M4287, Sigma)-treated mouse embryonic fibroblasts (feeder cells) (ASF-1201, AMS Biotechnology) in Fc medium supplemented with 2iLIF. After 48 hours blastocysts outgrowths were passaged by treatment with trypsin-EDTA for 20 minutes. After two passages in feeder cells, mouse ESCs were routinely cultured in gelatin-coated plates. Rex1::GFPd2 cells were kindly provided by Prof. Austin Smith, Wellcome - MRC Cambridge Stem Cell Institute.

Cloning

- PB TetO GFP-Podocalyxin Hygro: a GFP-Podocalyxin pDONR221construct (Shahbazi et al., 2017) was used to subclone GFP-Podocalyxin into a PB TetO hygro destination vector (kind gift of Dr. Jose Silva, MRC Cambridge Stem Cell Institute) using an LR clonase II (11791-100, Life technologies).
- PB rtTA3 Zeo: a pEnt L1L3-EF1a-rtTA3 (addgene plasmid #27106) was used as a template to amplify rtTA3 by PCR using the following primers:

attB1-rtTA3 FW:

5’-
GGGGACAAGTTTGTACAAAAAAGCAGGCTTCACCATGTCTAGACTGGACAAGA
GCAAAGTC-3’

attB2-rtTA3 RV:

5’-GGGGACCACTTTGTACAAGAAAGCTGGGTCTTACCCGGGGAGCATGTCAA-3’

The rtTA3 fragment containing attB sites was cloned into a pDONR221 vector using a BP clonase II enzyme (11789-100, Life Technologies), and further subcloned into a PB Zeo plasmid (kind gift of Jose Silva, MRC Cambridge Stem Cell Institute) using the LR clonase II.

- PB Bcl-2 Hygro: Bcl-2 was amplified by PCR from mouse cDNA using the following primers:

Bcl2 FW: 5’-ATGGCGCAAGCCGGGAG-3’

Bcl2 RV: 5’-TCACTTGTGGCCCAGGTATGCAC-3’

In a subsequent PCR amplification attB sites were added using the following primers:

attB1-Bcl-2 FW:

5’-GGGGACAAGTTTGTACAAAAAAGCAGGCTTCACCATGGCGCAAGCCGGGAG-3’

attB2-Bcl2 RV:

5’-GGGGACCACTTTGTACAAGAAAGCTGGGTCTCACTTGTGGCCCAGGTATGC-3’

The Bcl-2 fragment containing attB sites was cloned into a pDONR221 vector using a BP clonase II enzyme, and further subcloned into a PB Hygro plasmid (kind gift of Jose Silva, MRC Cambridge Stem Cell Institute) using the LR clonase II.

Cell transfection

E14 mouse ESCs were transfected using Lipofectamine 3000 (L3000001, Thermo Fisher Scientific) in agreement with the manufacturer’s recommendations. Briefly, 50.000 were cells seeded in a 24 well plate dish in Fc medium supplemented with 2iLIF the day prior to the transfection. Transfection was done with 0.3 µg of the plasmid(s) containing the gene(s) of interest and 0.2 µg of a plasmid expressing a PiggyBac transposase (kind gift of Prof. Jose Silva, MRC Cambridge Stem Cell Institute). Transfected cells were selected using the relevant antibiotics as outlined below:

- Doxycycline inducible GFP-PODOCALYXIN expressing mouse ESCs: cells were selected with 200 µg mL-1 of hygromycin B (10687010, Thermo Fisher Scientific) and 100 µg mL-1 of zeocin (ant-zn-1, Invivogen).

- BCL-2 overexpressing mouse ESCs: cells were selected with 200 µg mL-1 of hygromycin B. Experiments were performed with a mixed population of cells as individual clones were not picked.

H2B-GFP expressing mouse ESCs have been described elsewhere (Shahbazi *et al.*, 2017)

Live imaging

GFP-Podocalyxin expression was triggered by adding 1 µg mL^{-1} doxycycline hyclate (D9891, Sigma) to the medium. SYTOX Orange (S11368, Thermo Fisher Scientific) was used according to the manufacturer's instructions. Imaging was carried out on a Leica SP8 inverted confocal microscope (Leica Microsystem) using a Leica Fluotar VISIR 0.95 NA x25 objective. Time points were taken every 60 minutes between 24 and 96 hours after 2iLIF withdrawal with a z-step of 2.5 μm. Cells were imaged in a humidified chamber at 37 °C and 5% CO_2.

Immunostaining

Embryos and mouse ESCs were fixed in 4% Paraformaldehyde (11586711, Electron Microscopy Sciences) for 20 minutes at room temperature and permeabilized for 20 minutes in PBS 0.5% Triton X-100. Samples were incubated overnight at 4°C in blocking buffer (10% filtered fetal bovine serum, 0.1% Triton X-100 in PBS) with primary antibodies. Incubation with secondary antibodies was carried out for 2 hours at room temperature in blocking buffer. The following primary antibodies were used: Cleaved CASPASE-3 (9664S, Cell Signaling Technology) at 1:300, GM130 (610822, BD Biosciences) at 1:300, OCT4 (sc-5279, Santa Cruz Biotechnology) at 1:300, NANOG (ab80892, Abcam) at 1:300, CDX2 (MU392-UC, Launch Diagnostics) at 1:300, SOX17 (AF1924, R&D Systems) at 1:300, PODOCALYXIN (MAB1556, R&D Systems) at 1:500, BCL-2 (ab182858, Abcam) at 1:300, GFP (GF090R, Fine Chemical Products Ltd) at 1:500, COLLAGEN IV (AB769, Millipore) at 1:300. Conjugated secondary antibodies from Thermo Fisher Scientific were used at 1:500. F-ACTIN was visualized using Alexa Fluor488-phalloidin (A12379, ThermoFisher Scientific) or Alexa Fluor647-phalloidin (A22287, ThermoFisher Scientific) at 1:500. Nuclei were stained with DAPI (D3571, Life Technologies) at 1:500.

Imaging and Image analyses

Scanning was carried out on a Leica SP5 or SP8 inverted confocal microscope (Leica Microsystem) using a Leica HC PL APO CS2 40x or a Leica Fluotar VISIR 0.95 NA 25x objective. The software Fiji (Schneider et al., 2012) was used for image processing and analysis. Cell numbers in pre- and post-implantation embryos and in mouse ESC aggregates were manually counted in Fiji using the "Multi-point" tool. In E4.5 and E5.5 embryos all epiblast cells were counted by selecting individual nuclei across the different Z-planes. Quantifications shown in panels 1J, S1O-Q and 5K were

done on a single representative Z-plane. The apoptotic index was calculated by dividing the number of cleaved CASPASE-3 positive cells by the total number of epiblast cells in embryo analyses and by the total number of cells in ESC aggregates cultured in agarose. For the analysis of apoptosis in mouse ESC aggregates, the number of cleaved CASPASE-3 positive cells within the lumen was manually counted in Fiji. For the analyses shown in Figure S2E-F the total number of apoptotic cells in a representative single plane was counted, since aggregates cultured in naïve pluripotent conditions do not have lumens. For the classification of mouse ESC aggregates into different bins, for each experiment the area of individual aggregates in the maximum projection was measured in Fiji by manual drawing using the "Measure, Area" tool. Mouse ESC aggregates within the percentiles 1-33, 33-66 and 66-100 were considered small, medium and large respectively.

Statistical analyses

Statistical analyses were performed using GraphPad. Sample size for embryo-ESC chimera experiments was determined using power calculations for a χ^2 test (α=0.05, 1-β=0.95) using G*Power. In the rest of experiments sample size was determined based on previous experimental experience. Investigators were not blinded to group allocation and embryos were randomly allocated to control and experimental groups. Exclusion criteria for post-implantation embryos are stated in the text and summarised in Table S1 and S2. Qualitative data are presented as percentages of total events, contingency tables with raw event counts were analysed with a χ^2 test. Quantitative data are presented as mean ± S.D. and were analysed for normality with a D'Agostino-Pearson normality test. If passed, a two-tailed unpaired Student's *t*-test was used while for non-Gaussian distributions a Mann-Whitney-*U*-test was performed. To compare multiple groups an ANOVA test with a Tukey's multiple comparison test was performed. For linear correlation analyses, the Pearson correlation coefficient was computed.

FIGURE S1

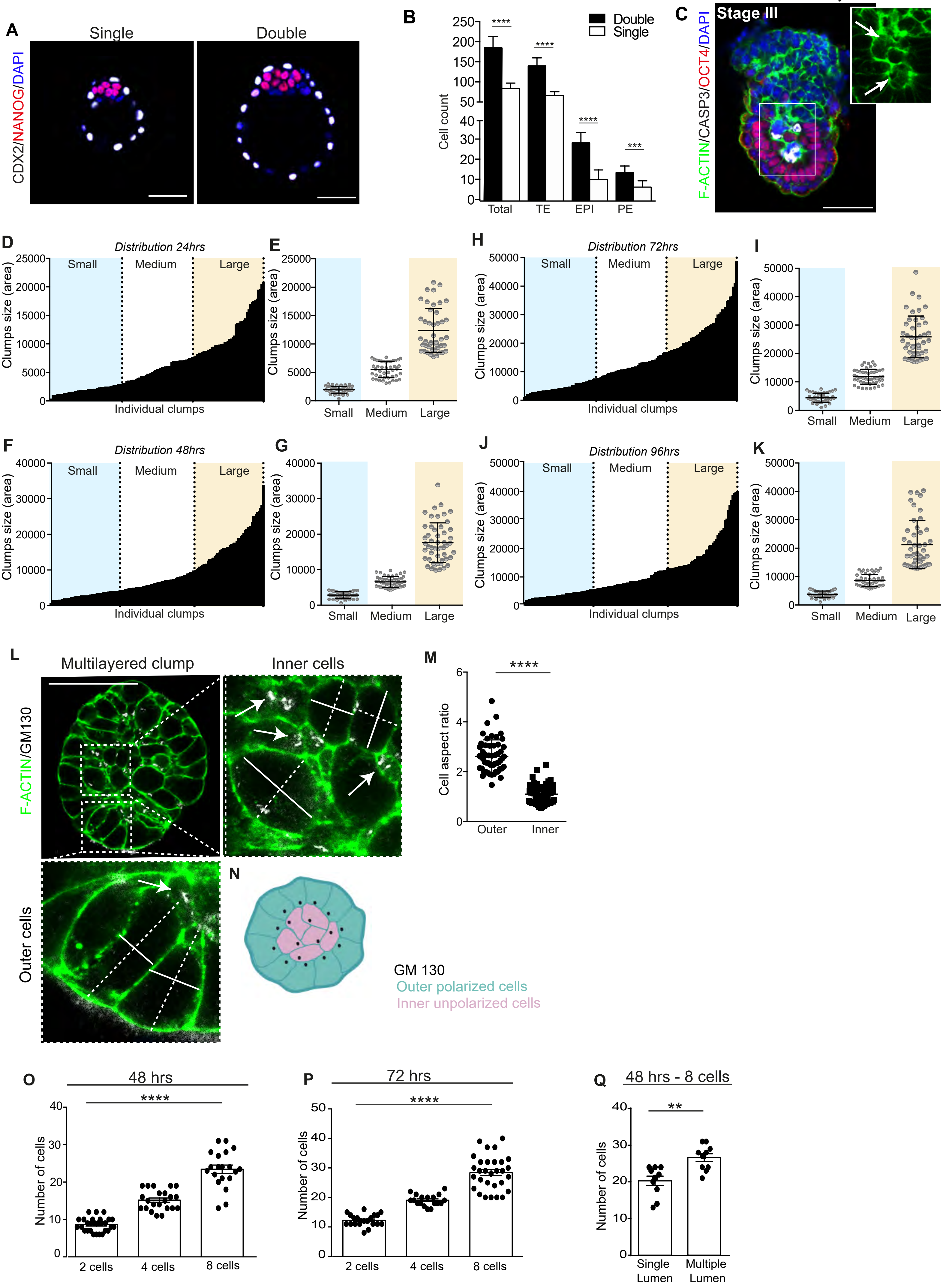
A
Single
Double
CDX2/NANOG/DAPI
B
Double
Single
Cell count
Total
TE
EPI
PE
C
Double Embryo
Stage III
F-ACTIN/CASP3/OCT4/DAPI
D
Distribution 24hrs
Small
Medium
Large
Clumps size (area)
Individual clumps
E
F
Distribution 48hrs
G
H
Distribution 72hrs
I
J
Distribution 96hrs
K
L
Multilayered clump
Inner cells
F-ACTIN/GM130
Outer cells
M
Cell aspect ratio
Outer
Inner
N
GM 130
Outer polarized cells
Inner unpolarized cells
O
48 hrs
Number of cells
2 cells
4 cells
8 cells
P
72 hrs
Q
48 hrs - 8 cells
Single Lumen
Multiple Lumen

FIGURE S2

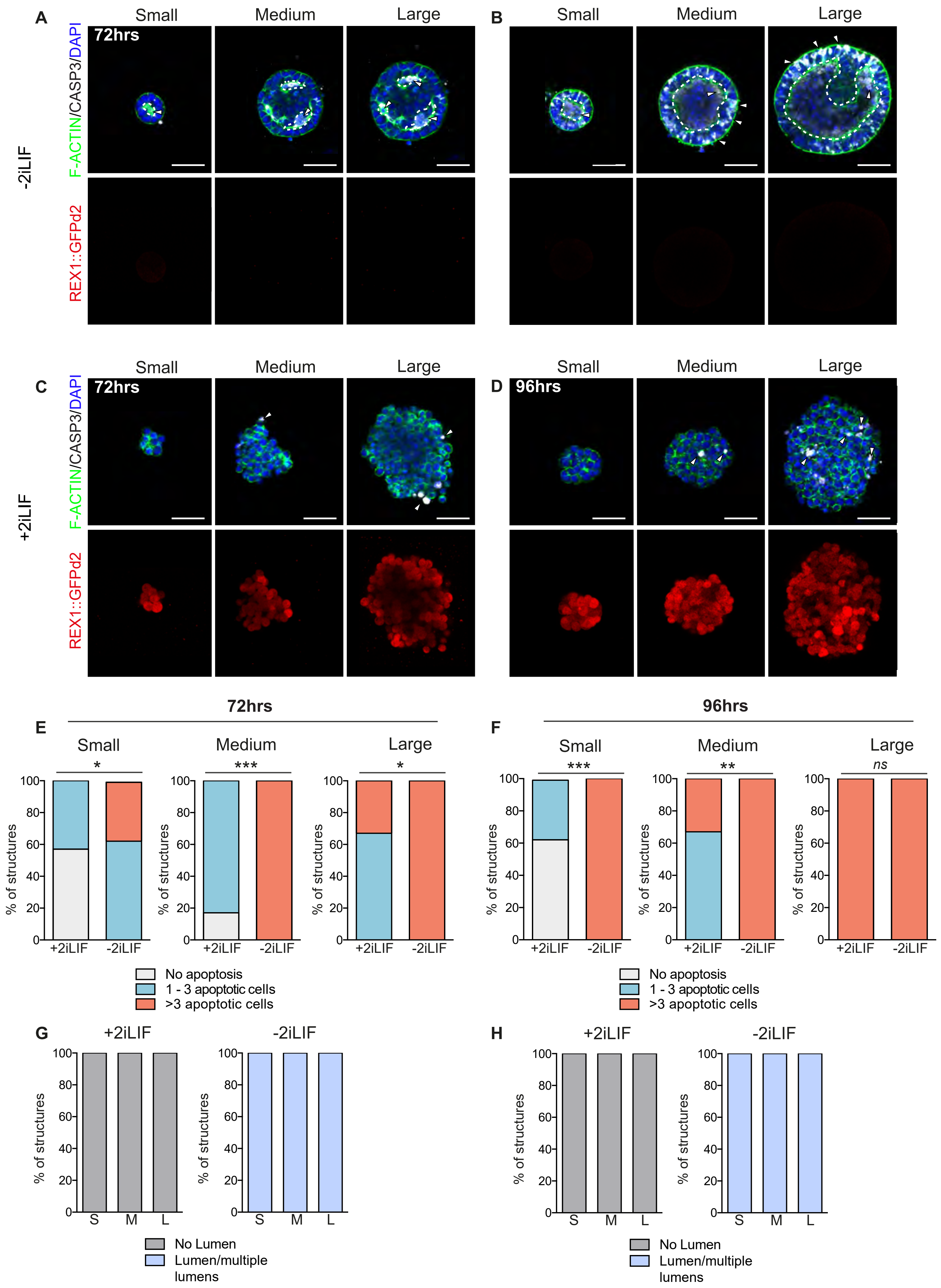

A
B
C
D
Small
Medium
Large
72hrs
96hrs
-2iLIF
+2iLIF
F-ACTIN/CASP3/DAPI
REX1::GFPd2
E
F
72hrs
96hrs
*
***
**
ns
% of structures
+2iLIF
-2iLIF
No apoptosis
1 - 3 apoptotic cells
>3 apoptotic cells
G
H
S
M
L
No Lumen
Lumen/multiple lumens

FIGURE S3

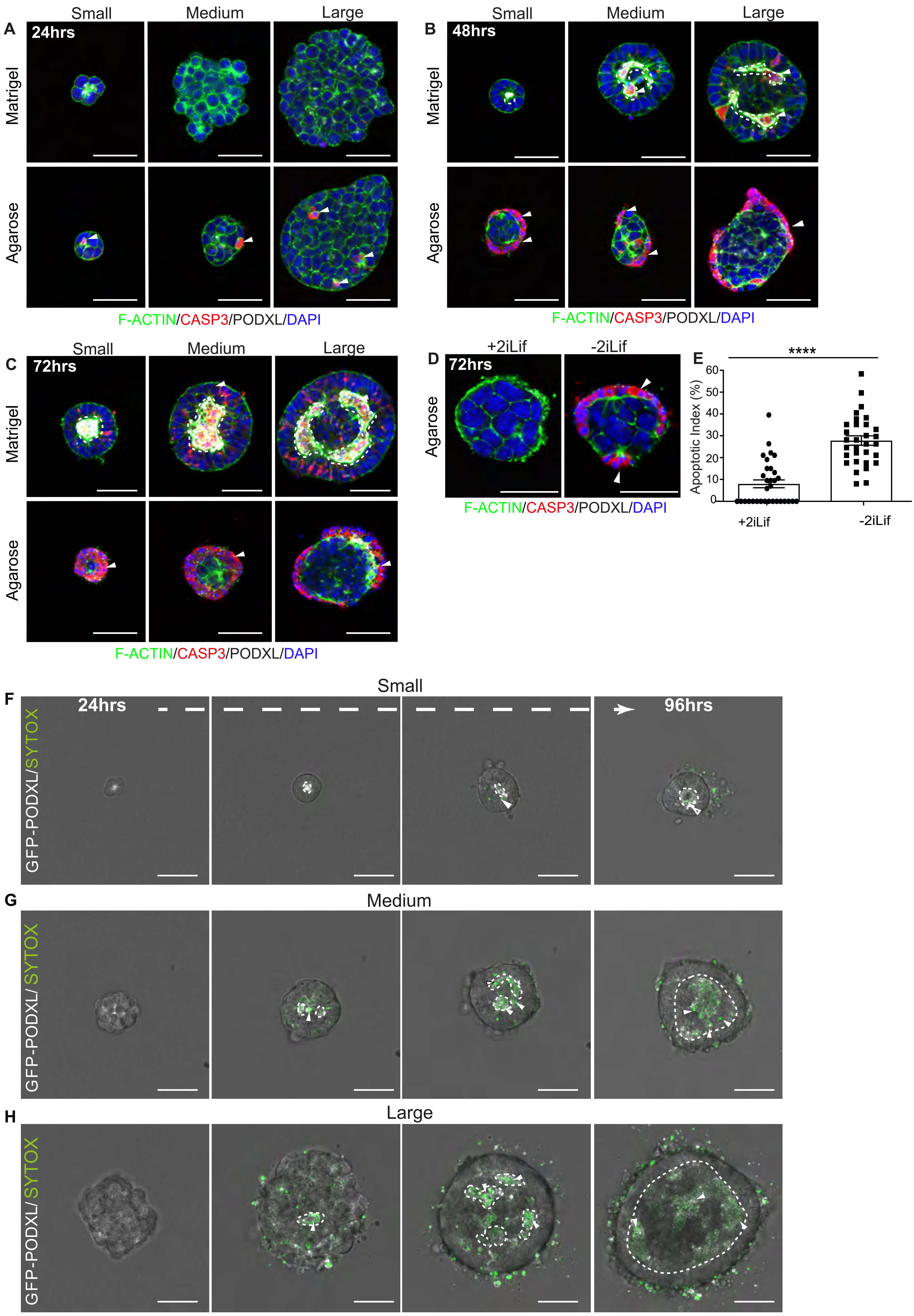

A
B
C
D
E
F
G
H
Small
Medium
Large
24hrs
48hrs
72hrs
Matrigel
Agarose
F-ACTIN/CASP3/PODXL/DAPI
+2iLif
-2iLif
****
Apoptotic Index (%)
0
10
20
30
40
50
60
96hrs
GFP-PODXL/SYTOX
GFP-PODXL/ SYTOX

FIGURE S4

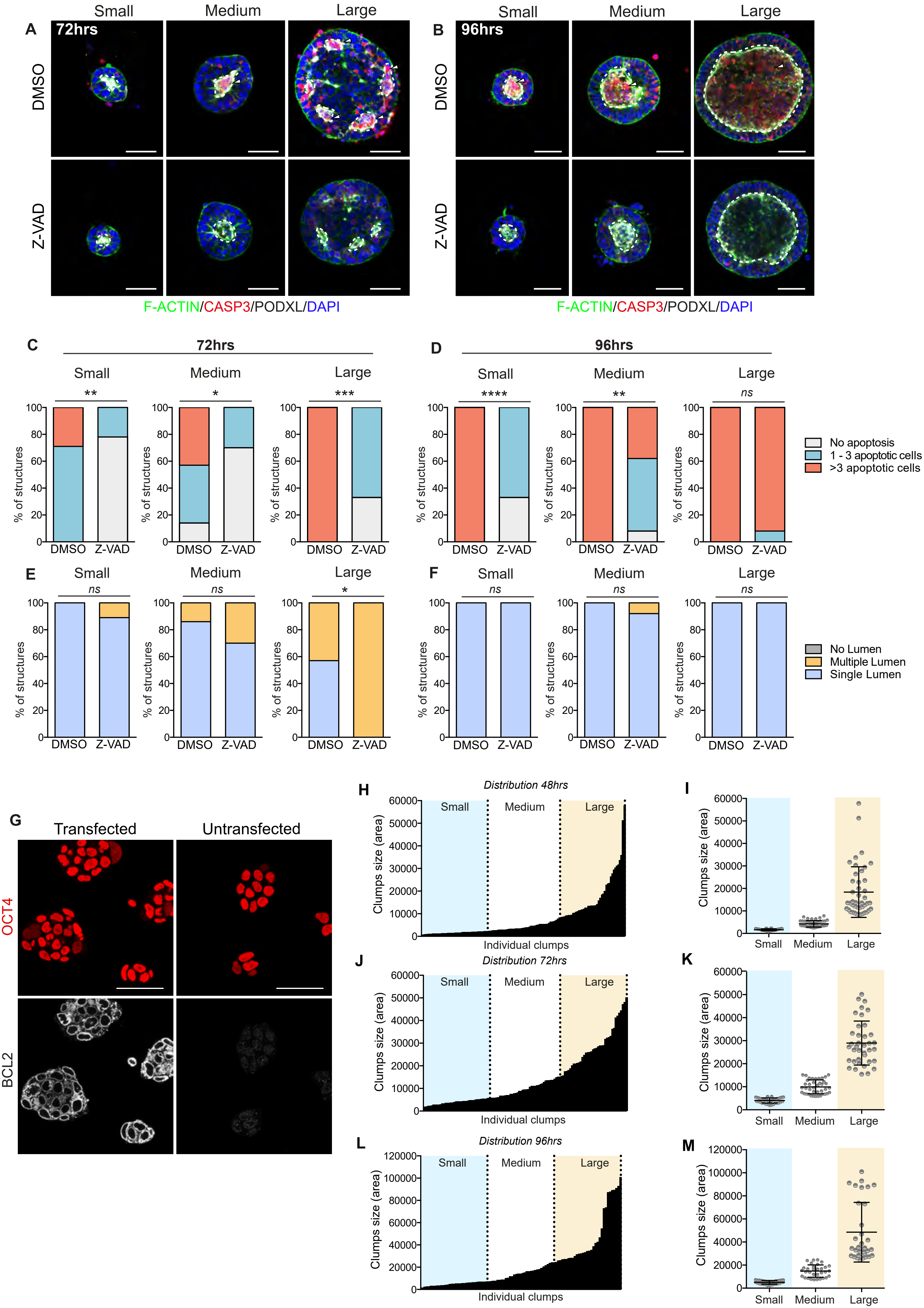
A
B
Small
Medium
Large
72hrs
96hrs
DMSO
Z-VAD
F-ACTIN/CASP3/PODXL/DAPI
C
D
E
F
Small
Medium
Large
**
*
***
****
ns
% of structures
DMSO
Z-VAD
No apoptosis
1 - 3 apoptotic cells
>3 apoptotic cells
No Lumen
Multiple Lumen
Single Lumen
G
Transfected
Untransfected
OCT4
BCL2
H
I
J
K
L
M
Distribution 48hrs
Distribution 72hrs
Distribution 96hrs
Clumps size (area)
Individual clumps
